# Who is disrupting whom? Examining the nighttime activity patterns of humans and their dogs

**DOI:** 10.1371/journal.pone.0358453

**Published:** 2026-10-07

**Authors:** Ming Fei Li, Lavania Nagendran, Christopher Kiru, Lauren Schroeder, David R. Samson

**Affiliations:** 1 Department of Anthropology, University of Toronto, Toronto, Ontario, Canada; 2 Department of Evolutionary Anthropology, Duke University, Durham, North Carolina, United States of America; 3 Department of Anthropology, University of Toronto Mississauga, Mississauga, Ontario, Canada; The University of Texas at El Paso, UNITED STATES OF AMERICA

## Abstract

Few studies have examined the nighttime dynamic between humans and dogs, but existing research suggests dogs can disrupt human sleep. In this study, we examined the nighttime activity of 25 Canadian participants, totaling 38 human-dog pairs using accelerometers and survey data. We used linear mixed effects models to assess the drivers of variability in both human sleep and dog nighttime activity, and a generalized linear model to assess the effects of demographic variables on self-reported sleep quality. None of the variables significantly affected objective sleep measures in humans or dog nighttime activity. Dog sleeping location did not affect human perceived sleep quality, but having multiple dogs was associated with poorer subjective sleep (p = 0.042). Human and dog nighttime activities were found to be significantly correlated. Additionally, we conducted a time series analysis to determine lagged correlations between human and dog activities within five minutes. Among the 61% dyads that showed correlation in activity states, human activity preceding dog activity (45%) occurred more often than dog activity preceding human activity (18%). In 36% of dyads, activity was synchronized within the same 1-minute period. Overall, co-sleeping (i.e., bedsharing) had no effect on sleep duration or efficiency in humans or nighttime activity in dogs. The only significant predictor we found was the presence of other dogs, which affected subjective but not objective human sleep quality, revealing a discrepancy between self-reported and objectively measured sleep. Both humans and their dogs impact the nighttime activity of one another, demonstrating our species’ inseparable and reciprocal relationship.

## Introduction

Dogs (*Canis familiaris*) and humans share an evolutionary history spanning at least 15,000 years [1]. Over this time, dogs developed numerous adaptations that have allowed them to cohabit closely with humans. Experimental studies exploring the social-communicative behaviors of dogs and socialized wolves show that dogs are more effective at interpreting human gestures [2–5]. Both dogs and humans show an increase in oxytocin concentration after tactile interaction and mutual gazing, which is not observed between humans and socialized wolves [6]. Furthermore, dogs are able to recognize human emotions [7]. These behavioral and physiological adaptations support the strong social bonds between dogs and humans.

Dog ownership can impact human physical and psychological health. Dog owners are more likely to engage in regular physical activity that contributes to better cardiovascular health compared to non-dog owners [8]. Pet ownership was also positively associated with the survival of myocardial infarction patients experiencing depression [9]. Though systematic reviews suggest that pet ownership can improve psychological health, the association is not always positive [10–13]. For instance, the stress and grief of losing a pet can have a negative psychological effect on owners [14]. Another potential negative effect of pet ownership is its impact on human sleep [15].

Interspecies co-sleeping, defined as bedsharing among humans and companion animals, is an extreme expression of multispecies entanglement [16]. More recently, with the advent of wearable devices for tracking sleep under natural conditions (home environments instead of laboratory settings), the research on co-sleeping in human and companion animals, particularly dogs, has become a topic of interest. Several studies have found co-sleeping with dogs can cause sleep disturbances in humans [17–19], although others found no significant effect [20]. Interestingly, some of these studies show a disparity between subjective and objective sleep measures where participants reported better subjective sleep even though objective sleep was more poor. For example, humans may experience increased sleep disruptions but still report better subjective sleep, which may be a result of psychological benefits of co-sleeping with dogs, reviewed in Andre et al. [21]. Studies exploring how co-sleeping affects the nighttime activity of dogs are limited but suggest human activity can influence dog activity [17].

The sleep-wake cycles of humans and dogs differ, with dogs exhibiting shorter, polyphasic sleep patterns, which means multiple sleep bouts throughout the day [22]. This misalignment of sleep patterns between humans and their dogs could result in co-sleeping contributing to increased human sleep disruptions, as suggested by previous studies [17], although findings from other studies are mixed [20]. Additionally, the effects of co-sleeping on dogs’ nighttime activity are less documented. This study aims to explore the bidirectional effects of co-sleeping on human sleep and dog nighttime activity.

In this study, we use accelerometry and survey data to first explore how different demographic and household characteristics affect sleep in humans and nighttime activity in dogs. Second, we assess the correlation between human and dog activities during human sleep periods. First, we hypothesize that human and dog co-sleepers would negatively impact objectively measured sleep duration and quality in humans due to increased nighttime disturbances. Second, we hypothesize that proximity and number of dogs would impact subjective sleep quality, but the direction of these effects is undetermined. And finally, we expect that dog and human nighttime activities would be near-synchronous.

## Materials and methods

### Ethical statement

Ethical approval for this study was obtained through the University of Toronto Human Research Ethics Board (Protocol #40107) and Animal Care Committee (Protocol #20012651). Methods are in accordance with the guidelines and regulations set forth by the University of Toronto, and written informed consent was collected from human participants prior to data collection. Human participants received monetary compensation for their involvement upon completion of data collection.

### Recruitment

Participants were recruited through word-of-mouth referrals and poster advertising on social media. We recruited human participants (over the age of 18) and their dogs who lived in or near Ontario. To minimize breed-level activity differences [23], we recruited huskies and malamutes between two to 12 years of age. We only recruited huskies and malamutes because this study was part of a larger project comparing activity of dogs and wolves, and these breeds are descended from an ancient lineage of Siberian dogs that dates back 9,500 years [24]. We excluded dogs with owner-reported medical issues that might influence their activity patterns. Recruitment took place from August 2, 2021, to October 31, 2021, and we recruited 25 humans and 38 dogs, as some humans had multiple dogs that participated in the study (Table A in S1 File).

Activity data was collected from the individual who identified as the primary caretaker of their dog(s). At the end of the four-week study period, each participant filled out a questionnaire to provide demographic, behavioral, and sleep-related information about themselves and their dogs (see S2 File). Questions included information about their sleeping arrangements and possible sleep disturbances, how many individuals were in the household, whether they co-slept with another person, distance from dogs while sleeping, and other relevant information. Participants did not need to co-sleep with their dogs to be enrolled. If a household had multiple dogs that met the criteria (desired age, breed, and health status), activity was collected from all dogs.

### Data collection

We used the CamNtech MotionWatch 8 actigraphy sensor (a form of accelerometry) to record activity in humans and dogs for approximately four weeks between November 2, 2021, to December 9, 2021. Each sensor contains a tri-axial microelectromechanical-system (MEMS) accelerometer that detects acceleration along three orthogonal (X, Y, Z) axes and records the summation of these movements as MotionWatch activity counts per 1-minute epoch, which was the sampling interval used for this study. Humans wore the accelerometer on their non-dominant hand around their wrist, and dogs wore a nylon collar with the sensor attached; similar methods have been used in other dog studies [25,26]. Due to the lockdown measures during the COVID-19 pandemic, materials were shipped to participants who were instructed to attach the accelerometer to themselves and to place the collar on their dogs. Wrist-worn actigraphy has been widely used in studies of human sleep [27] and has been validated against polysomnography [28]. At this time, the MotionWatch 8 device has not been validated for classifying physiological sleep/wake states in dogs; therefore, in this study, we use the terms “inactivity” and “activity” to describe dog activities. Although direct behavioral observations were not collected, our analyses examine broad rest-activity patterns, for which accelerometry is well established [17,29,30]. Notably, low or zero movement counts from triaxial accelerometry have predicted behavioral sleep in dogs with high sensitivity and specificity when compared with concurrent behavioural observations [31,32].

Once the data collection period was complete, the accelerometers were removed and shipped back to the researchers. The raw activity data were downloaded onto the MotionWare software (version 1.2.23), and each nightly human sleep period was visually identified and coded by author MFL as the time between “Lights Out” (characterized by a notable decrease in activity) and “Got Up” (characterized by a marked and steady increase in activity)—for more details, see Li et al. [33]. We used this human sleep period for all nighttime analyses of human and dogs.

In addition to the questionnaire, participants also filled out the Pittsburgh Sleep Quality Index (PSQI), a questionnaire to assess self-reported sleep quality over the past month [34]. The PSQI composite Global Score takes into consideration seven separate components: subjective “sleep quality,” “sleep latency,” “sleep duration,” “habitual sleep efficiency,” “sleep disturbance,” “use of sleeping medication,” and “daytime dysfunction.” Higher Global Scores indicate poorer sleep quality, with the minimum and maximum possible scores ranging from 0 to 21.

### Data processing and analyses

We excluded periods of missing data (i.e., non-wear time), which were visually identified from the raw data as consecutive zero activity counts lasting more than 30 minutes. We also excluded all dates when humans were apart from their dogs—as reported retroactively in the post-study questionnaire or communicated to the research team during the study period; see Table A in S1 File for reasoning for each dyad (human-dog pair). Two dyads (CAN095-CAH037; CAN065-CAH020) were excluded from the analyses using accelerometry data due to incomplete data—a total of 36 human-dog dyads were analyzed (note that some humans with more than one dog contributed to more than one dyad). All 25 human participants were included in the subjective sleep analysis since all completed the required questionnaires.

#### Analysis 1. Human nighttime sleep.

We fitted separate linear mixed-effects models (LMM) with the following objective sleep measures as the response variables: Total Sleep Time (TST), which is the total time spent in sleep according to the epoch-by-epoch wake-sleep categorisation, and Sleep Efficiency (SE), which is the total time spent in sleep expressed as a percentage of the entire sleep period. The three main predictors were: presence of other dogs (binary: yes/no; no as reference category), presence of human co-sleepers (binary: yes/no; no as reference category), and whether dogs slept on the bed (categorical: no/sometimes/yes; no as reference category). For humans who had more than one dog, we used the sleep location of the dog that slept the closest (i.e., if one of their dogs slept on the bed but others did not, we coded whether dogs slept on the bed as “yes”). We included human self-reported gender (binary: man/woman; woman as reference), age (continuous, standardized), and Body Mass Index, or BMI (continuous, standardized), as covariates and human ID as a random effect in the model to account for repeated observations per individual. We calculated BMI using the standardized formula: weight (kg) divided by height squared (m^2^). After specifying parameters of the LMM, we used the model selection tool “dredge” to generate candidate models with all possible combinations of predictors. We then averaged across models with ∆AICc < 10 following established recommendations [35]. We report the full average coefficients and standard errors [36]. Model averaging is useful when there are numerous predictors, as it accounts for model uncertainty and reduces the risk of relying on a single model. Instead, this approach averages across a subset of well-supported models. There was no issue of multicollinearity across predictor variables as all variance inflation factors (VIF) were below 5.

To examine if the presence of other dogs, the presence of human co-sleepers, and whether dogs slept on the bed influenced subjective sleep quality, we constructed a Generalized Linear Model (GLM) with PSQI Global Score as the response variable and the three variables above as the predictors. We ran a “base” model where we only included human gender, age, and BMI as the predictors and none of them were significant. Thus, we did not include them in our final model due to limited statistical power.

#### Analysis 2. Dog nighttime activity.

Dog total nighttime activity was the response variable and calculated as the sum of all activity counts during the human sleep period. We fitted a LMM with the presence of other dogs, whether dogs slept on the bed, number of humans in the household (continuous, standardized), and number of times fed per day (categorical: 1/2/ > 2 times per day; > 2 as reference category). We also included dog age (continuous, standardized), weight (continuous, standardized), sex as covariates and dog ID as a random effect. Due to dogs’ total nighttime activity being positively skewed, we log-transformed the response variable in the model [37]. We used the same model averaging method described above.

#### Analysis 3. Association between human and dog nighttime activities.

We first wanted to see whether human and dog nighttime activities were correlated. We constructed a LMM with total human total nighttime activity as the response variable, their corresponding dog’s total nighttime activity as the predictor, and human ID as a random effect.

Then, we performed a time series analysis to explore the correlation between dog and human activity states throughout the sleep period. First, we time-matched dog and human data for each night’s sleep period. Second, we assigned data to binary activity states—human epochs classified as “wake” were coded as “1” and “sleep” as “0.” For dogs, epochs with activity counts equal to 0 (i.e., inactive state) were coded as “0,” and epochs with activity counts greater than 0 (i.e., active state) were coded as “1.” Third, we calculated Yule’s Y to assess lagged associations between human and dog activity states for lags from −5 to +5 minutes. Negative lag values represent human activity preceding dog activity, while positive values indicate human activity following dog activity. Yule’s Y is a measure of association between binary variables, ranging from −1 (perfect negative association) to +1 (perfect positive association), with 0 indicating no association. Fourth, we used boxplots to visualize each dyad’s distribution of Yule’s Y values across lags −5 to +5 across all nights (Fig A in S1 File). Fifth, we visually assessed the presence of a peak, defined as a continuous series of at least three adjacent boxplots whose Yule’s Y interquartile range did not overlap zero, and which exhibited an upward trend followed by a downward trend. We also identified the corresponding lag time and recorded the median Yule’s Y at the peak.

All statistical analyses were performed in R Studio (v.2026.01.0 + 392; R v. 4.5.2) for Mac OS X [38]. We used R package “*lme4*” for fitting LMMs [39], “*performance*” for checking multicollinearity [40], “*MuMIn* for model averaging [41], and “*DHARMa*” for model diagnostics [42], and MuMIn for model averaging [43]. Overall model significance was determined by comparing the final model with the intercept-only model using a likelihood ratio test. All statistical tests were two-tailed, with alpha set to 0.05 for significance.

### Data availability

All data and code used to process and analyze data are publicly available on OSF (osf.io/7dt3c/).

## Results

A total of 25 human (21 women, 4 men) and 38 dog participants (15 female, 23 male) were recruited for the study. Humans’ mean Total Sleep Time was 6.23 h (± 0.92), Sleep Efficiency was 79.31% (± 4.33), and PSQI Global Score was 6.84 (±3.72; range: 2–16). Out of 25 human participants, 13 (52%) lived with more than one dog and 13 (52%) reported that their dog always slept in the room. Regarding co-sleeping, specifically bedsharing arrangements, 16 participants (64%) reported co-sleeping with another human, four (16%) reported that they always co-sleep with their dog, 10 (40%) reported sometimes co-sleeping, and 11 (44%) indicated that they never co-sleep with their dog. In our analysis, we assigned the 11 participants who never co-slept with their dog(s) as the control group (reference category). For full descriptive summaries of survey responses, see Table 1.

**Table 1:** Summary of human and dog demographic characteristics and summary of survey responses. The mean (± SD) for continuous variables and the ratio (percentage) for categorical variables are presented.

Human characteristics	
Gender	Women: 21 / 25 (84%) Men: 4 / 25 (16%)
Age (years)	38.9 ± 11.9
Weight (kg)	73.4 ± 13.8
Height (cm)	168.3 ± 7.9
BMI	26.01 ± SD 5.27
Num. household members	2.4 ± 1.4
Presence of human co-sleepers	Yes: 16 / 25 (64%) No: 9 / 25 (36%)
Presence of other dogs	Yes: 13 / 25 (52%) No: 12 / 25 (48%)
Dog sleeps in room	Yes: 13 / 25 (52%) Sometimes: 5 / 25 (20%) No: 7 / 25 (28%)
Dog sleeps on bed	Yes: 4 / 25 (16%) Sometimes: 10 / 25 (40%) No: 11 / 25 (44%)
Sleep interrupted by dog	Yes: 12 / 25 (48%) No: 13 / 25 (52%)
Feel safer with dog	Yes: 18 / 25 (72%) No: 7 / 25 (28%)
**Dog characteristics**	
Sex	Female: 15 / 38 (39%) Male: 23 / 38 (61%)
Age (years)	4.8 ± 2.5
Weight (kg)	29.9 ± 11.2
Feeding (/day)	1: 8 / 38 (21%) 2: 20 / 38 (53%) >2: 10 / 38 (26%)

### Analysis 1. Human nighttime sleep

For full model results, see Tables B and C in S1 File. In the LMM (n = 777 nights) examining drivers of human nighttime sleep variability, none of the main predictors had a significant effect on Total Sleep Time and Sleep Efficiency (Table B in S1 File). In the GLM for PSQI responses (n = 25 participants), we found that presence of other dogs had a significant positive effect on the Global Score (β = 0.321, p = 0.042, 95% CI [0.014, 0.633]; in other words, having more than one dog was associated with worse subjective sleep quality (Fig 1). It is important to note that when comparing to an intercept-only model, the PSQI model was not significantly better (n = 25, p = 0.116).

**Fig 1:**
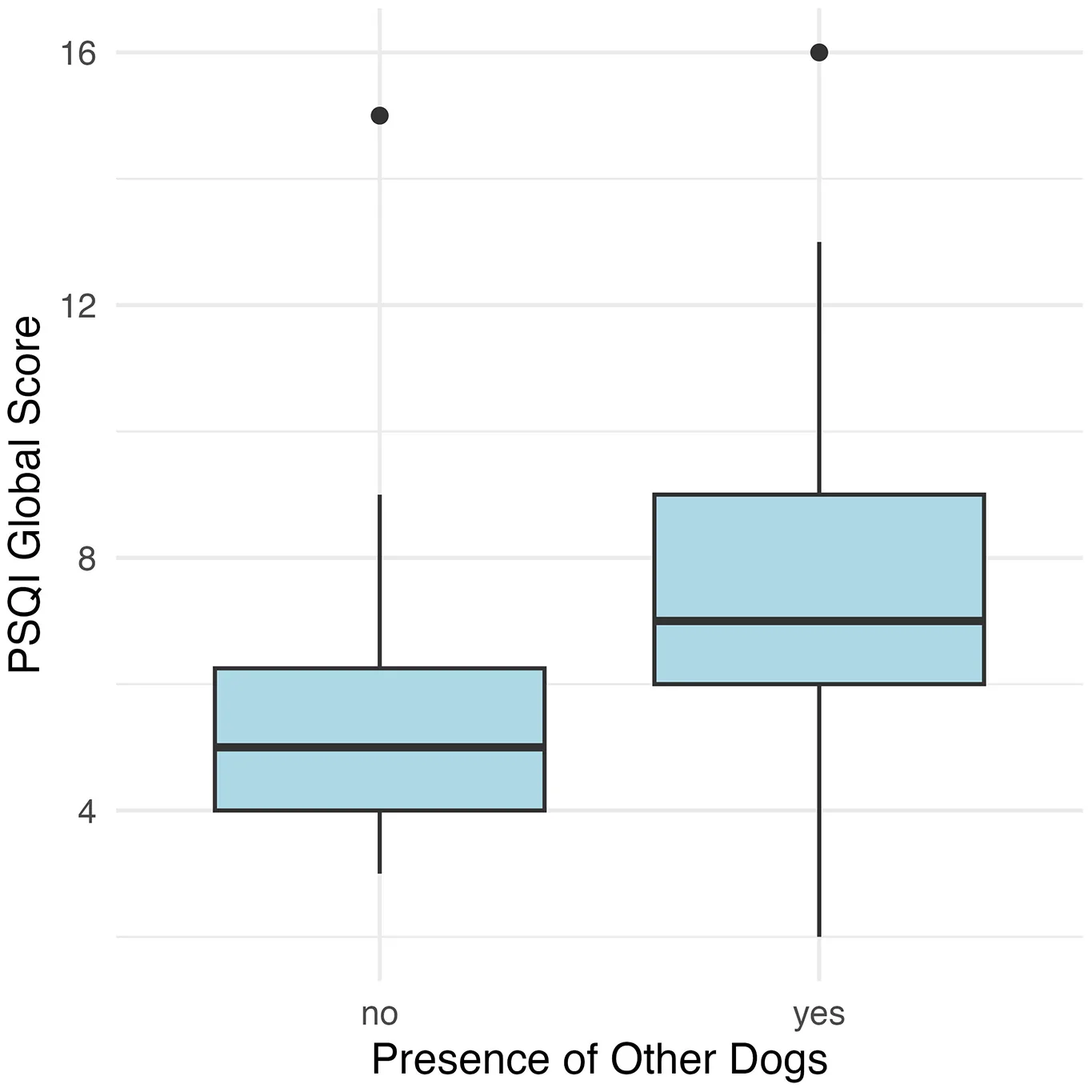
Boxplots comparing the PSQI Global Score (higher scores indicate poorer sleep quality) of humans who had (yes) or did not have (no) other dogs.

### Analysis 2. Dog nighttime activity

In the LLM (n = 1,093 nights) looking at drivers of dog nighttime activity variability, none of the predictors in the model had a significant effect on dog nighttime activity (Table C in S1 File).

### Analysis 3. Association between human and dog nighttime activities

In the LLM exploring the association between human and dog nighttime activities, dog total nighttime activity was significantly positively correlated with human total nighttime activity (β = 0.024, p = 0.003, 95% CI [0.008, 0.040]; overall model: n = 1,093, p = 0.003).

We examined Yule’s Y boxplots for 36 human-dog dyads to determine lagged cross-correlation between human and dog nighttime activity states from lags −5 to +5 minutes to see whether there was detectable association in activity states within a 5-minute window. (Fig A in S1 File). For example, in a given human-dog dyad, if we detected a positive Yule’s Y peak at lag time −1, it means that human activity at t-1 correlated with dog activity at t0 (i.e., dog activity was most strongly positively associated with human activity from one minute ago). We detected a positive Yule’s Y peak (i.e., positive association between human and dog activity states) in 22 out of 36 dyads (61.11%) while 14 out of 36 dyads (38.89%) did not have a peak. Of the 22 dyads with a peak, four (18.18%) had a peak at a positive lag (dog activity preceded human activity), eight (36.36%) had a peak at 0 lag (dog and human activity occurred within a 1-minute interval), and ten (45.45%) had a peak at a negative lag (human activity preceded dog activity). Among the three dyads that *always* co-slept on the same bed in which we identified a peak, one had a peak at 0 lag, two had a peak at a negative lag. Among the eight dyads that *sometimes* co-slept on the same bed in which we identified a peak, five had peaks at 0 lag and three had a peak at a negative lag. Among the eleven dyads that *never* co-slept on the same bed in which we identified a peak, four had peaks at a positive lag, two had peaks at 0 lag, and five had a peak at a negative lag.

## Discussion

In this study, we used accelerometry and survey data to measure activity in humans and their dogs. We did not find evidence that bedsharing co-sleepers (both human and dog) had any effects on humans’ objective sleep duration or quality, nor did human co-sleepers affect dogs’ nighttime activity. Thus, we found no support for our first hypothesis. Based on self-reported sleep quality (PSQI), humans with multiple dogs in the household had worse sleep quality than humans with only one dog. Our second hypothesis was supported since we found that the number of dogs negatively impacted subjective human sleep. When assessing the association between human and dog nighttime activities, we found that they were positively correlated, meaning that nights during which there were higher counts of dog activity corresponded to higher counts of human activity. Furthermore, our time-series analysis indicates that in majority of the dyads, there was evidence of tight coupling between human and dog activities within a 3-minute time interval (lag −3 to lag + 2 minutes), supporting our third hypothesis that dog and human activities would be positively correlated.

### Co-sleeping did not affect objective sleep duration or efficiency in humans

None of the variables we expected to disrupt objectively measured sleep in humans (e.g., presence and number of human and dog co-sleepers) were significant in our analysis. Actigraphy studies show that co-sleeping in adult humans can result in increased sleep disruption in one or both individuals [44,45]. Drews et al. [46] did not find that co-sleeping had any significant association with sleep efficiency, which aligns with our finding that co-sleeping may not affect overall sleep duration or quality. Additionally, none of the three covariates (gender, age, BMI) were significant drivers of human nighttime sleep variability. Taken together, our null findings imply that co-sleeping with humans or dogs did not affect sleep duration or efficiency across different demographics, but a larger and more balanced sample group is needed. Currently, no actigraphy studies have examined whether co-sleeping with pets has different effects on different gender and age groups. It will be interesting to see whether the effect of pet ownership mirrors the baseline sleep differences typically found between genders [47,48] and age-groups [49]. It is important to highlight that since we only recruited dog owners, we did not have a comparative group consisting of humans without dogs and therefore could not assess whether the presence of dogs affected human nighttime sleep. One study found no significant differences between dog owners and non-dog owners in self-reported sleep duration or sleep quality [50], while another study found that dog owners who regularly walked their dogs had greater light sleep than non-dog owners but did not significantly differ in deep sleep or nighttime awake time [51]. Interestingly, we did find that the number of dogs in the household affected perceived sleep quality.

### Humans with multiple dogs reported poorer subjective sleep quality

Humans who had more than one dog reported worse sleep quality based on PSQI scores than those with only one dog. Contrary to what we found, Chin et al. [15] found that the number of pets in the household was not associated with perceived sleep quality, but instead was associated with greater insomnia severity. Hoffman et al. [52] also found no significant association between the number of dogs in the household and subjective sleep quality. The literature on how multiple pets might influence perceived sleep quality is scarce; further research on this topic is needed, such as whether the number of pets in the household has an additive effect.

In a nationally representative sample of US adults, co-sleeping with pets was found to correlate with poorer perceived sleep quality only among dog-owners, and not cat-owners [15]. Many studies have found no discernible effect of pet ownership on self-reported sleep quality based on PSQI. An online Swedish study found that dog ownership was not associated with any indicator of poor sleep quality in self-reported sleep outcomes [50]. A national online survey in Australia found that humans who co-slept with pets did not differ in self-reported sleep length or likelihood of waking up due to a disturbance than those who did not co-sleep with pets [53]. Additionally, co-sleeping with pets did not affect subjective sleep quality among children [54] or adolescents [20].

Many studies have noted the discordance between objective and subjective sleep quality among pet owners. A review by Andre et al. [21] found that humans reported better subjective sleep quality when sharing a bed with both human and dog co-sleepers, despite worse objective sleep measures. Similarly, even when actigraphy measures indicated that dogs increased sleep disturbances, humans did not report their sleep quality to suffer due to nighttime disruptions by dogs [17,19]. This may reflect the psychological benefits (e.g., feeling safe) of co-sleeping with dogs [17,21].

When participants were directly asked whether and how their sleep was affected by dogs, our survey responses indicated a mixed perception—half of the participants thought that dogs interrupted their sleep, while the other half did not. These findings support previous research results, which also found mixed evidence regarding perceived sleep disruptions due to pets. Krahn et al. [55] found that 20% of pet owners thought that pets were disruptive, while 41% thought pets were unobstructive or beneficial to sleep. Hoffman et al. [56] found that 33% of adult women reported sleeping better when in body contact with their dog, 26% reported sleeping better without body contact, and 40% did not think their sleep was affected by contact with their dogs.

In summary, we did not find that dogs’ sleeping location affected humans’ perceived sleep quality, supporting findings from Patel et al. [18] but counter to Hoffman et al. [52]. Interestingly, we found that having multiple dogs was associated with worse subjective sleep, which contributes to the limited literature that currently exists for this topic [15,52].

### No significant drivers of dog nighttime activity variability

Similar to our human sleep models, our dog nighttime activity model showed no significant effects for the presence of other dogs, the presence of other humans, and the dogs’ sleeping location on nighttime activity. Li et al. [26] previously found that nonworking sled dogs who slept in kennels with two versus three roommates did not differ in their nighttime activity. Hoffman et al. [17] assessed the actigraphy data of bedsharing human-dog dyads and found that dogs were generally more active than humans at night, and human movement leading to dog movement was less likely than dog movement leading to human movement. Although this study only measured activity in dogs that co-slept on the bed, their findings show that dog activity may be less sensitive to human movement during human sleep periods.

In studies of dogs that found biological variables to affect daytime activity (e.g., sex, age), these effects were no longer significant during the nighttime [26,29]. These findings are consistent with our results that biological variables had no effect on nighttime activity in our dog group. One exception is Zanghi et al. [57], who found a negative age effect on nighttime activity in beagles—this study included a wider age range including a greater sample of senior dogs (11–14 years). The oldest dog in our study was 11 years old, with the average age being 4.8 years. Therefore, our study may not capture potential changes in activity among older dogs.

Research by Zanghi et al. [30,57] found that the number of feedings affects the nighttime activity of kennel-housed dogs—nighttime activity was higher in dogs fed twice daily compared to once daily. Contrarily, our study did not find an effect on the number of feedings and dog nighttime activity. This discrepancy may be a result of the flexibility of feeding times and quantity across households in our study, which we did not collect data on.

It is well-established that dog activity is highly entrained to human activity [29,58,59]. When human activity levels remain low during nighttime sleep, these low activity levels are also reflected in the majority of dogs who are mostly asleep or resting. It is plausible that during these periods of consistent low activity in dogs, the effects of all variables we included in the model are negligible (i.e., not enough variation across individuals).

### Human activity tended to precede dog activity

Despite the relatively lower activity counts during nighttime sleep periods, we found that human and dog nighttime activities were positively correlated, albeit with a small effect size. This positive correlation between human-dog nighttime movements was also reported by Hoffman et al. [17]. Once we take a closer examination into activity association within human-dog dyads, some noticeable trends emerge. Approximately 39% of dyads showed no correlation between the activity states of humans and dogs. The majority (~61%) of the dyads showed a significant association between human and dog nighttime activity, and among these, a greater proportion (~45%) showed that human activity preceded dog activity within a five-minute interval, and a smaller proportion (~18%) showed that dog activity preceded human activity. Our findings are not in line with previous time-series approaches to investigating human-dog dyadic movements. Smith et al. [19] found that dog movement, up to 2.5 minutes prior, preceded human movement. Similarly, Hoffman et al. [17] found that the odds of dogs influencing human movement were higher than those of humans influencing dog movement. Questionnaire responses suggest that humans did not feel like their dogs were interrupting their sleep. Out of 25 participants, the vast majority did not feel their sleep was interrupted by dog barking (88%) or noise caused by dog movements (84%). When we asked participants how often they wake up at night due to their dog(s), 14 participants reported zero times, nine participants said one or two times, and two participants said more than two times.

One potential reason for why human activity occurred prior to dog activity could be that human nighttime activity is indicative of poorer sleep, characterized by sleep disruptions. Socioeconomic instability [60,61] and lack of social support [62,63] have been linked to poorer self-rated sleep quality. Considering that we collected data during a period of heightened stress (i.e., lockdown during the COVID-19 pandemic), humans in our study may have experienced more disrupted sleep, characterized by increased nighttime activities, which then disturbed their dogs and contributed to subsequent dog activity. A systematic review assessing the impacts of the COVID-19 pandemic on actigraphy-measured sleep found that the pandemic was associated with multiple indicators of poor sleep quality, such as decreased sleep efficiency and increased frequency and duration of nighttime awakenings [64]. This is potentially reflected in the average PSQI Global Score of 6.84 among our human participants—for reference, scores 5 and above indicate poor sleep.

In our study, ~ 36% dyads exhibited activity synchrony within a 1-minute epoch, meaning that within this interval, we could not determine which individual was leading the activity bout. Future work should collect data at higher frequency (i.e., collect data at 5- or 10-second epochs) to tease apart the precise time frame during which we see human-dog activity synchrony. Where feasible, pairing actigraphy with video recording and noise monitoring could provide more detailed insight into the divers of nighttime activity in humans and dogs.

Where dogs slept relative to humans matters. We did not see any clear trends among dogs with different co-sleeping locations and the presence of a Yule’s Y peak (i.e., whether there was a significant association between dyadic activity states). Among dyads with peaks, in all four cases where human activity was driven by dog activity, the dogs were not on the bed. In two out of three cases where dogs slept in the bed, their activity was driven by human activity, and activities were synchronized in the other case. This finding implies that dogs who were at a greater distance from humans were more likely to drive human nighttime activity. In another study [52], dogs’ sleeping location did not affect subjective sleep quality, but owners whose dogs slept on the bed reported feeling less tired than those whose dogs did not sleep on the bed. However, Patel et al. [18] found that humans who co-slept with dogs on the bed had lower actigraphy-measured sleep efficiency than those with dogs who slept in the room. Future research should further investigate this relationship—one way to do this could be asking participants to keep a diary to record nightly sleeping arrangements (e.g., sleep location for dog(s) and human(s), recalled nighttime waking causes). Participants in this study only completed the questionnaire once at the end of the four-week study period, which likely introduced some recall bias—keeping a diary would minimize this bias. Future work should also account for sleep environment characteristics, such as bed size, which may influence human and dog nighttime movements.

Investigations into human and dog activity and sleep have been and will continue to be subject to the inevitable influence of cultural factors and individual variations that are difficult to capture in statistical models [65,66]. More nuanced approaches are clearly needed, such as a combined approach of both observational and actigraphy data.

### Limitations

There are several study limitations that warrant discussion. First, our results are derived from a small sample of human and dog participants in southern Ontario, where there was a gender imbalance (more women volunteered than men). As such, our findings may not be generalizable to other demographic and geographic groups. Second, data collection occurred while COVID-19 lockdown measures were in place. Thus, there was substantial flexibility and variability in work arrangements that would have affected the routines of both human and dog participants. Although all participants were the primary, or co-primary, caretakers of their dog, we did not account for the activity of other humans in the household. Third, we set the MotionWatch 8 to tri-axial “Mode 3,” which was the setting recommended for detecting physical activity and movement along three axes. However, for this device, only “Mode 1” (summation of movement along one axis) has been validated against polysomnography for quantifying sleep in humans. Since the tri-axial mode was more sensitive to movement, we set the sleep analysis function to “medium” sensitivity rather than “high” sensitivity used for Model 1. In this study, we restrict our analyses to within-group comparisons, so we believe our results remain sound. Fifth, our study only examined the activity of huskies or malamutes, and breeds may vary in their activity patterns [23]. Therefore, our findings are not generalizable across all dog breeds. However, by accounting for breed-level differences, which is seldom done in other human-dog co-sleep studies, we are better able to assess variables of interest.

One major limitation of this passive, remote-monitoring study was the absence of direct behavioral observations which prevented us from identifying noise disturbances (e.g., snoring) or the specific behaviors that generated the movement captured by the accelerometer. Some human movements may have generated more noise and been more likely to disturb dogs (e.g., leaving the room to use the toilet) than others (e.g., moving in bed). Furthermore, the presence of any dog movement that was detected by the accelerometer (activity counts > 0) was coded as “active” state. This approach may have overestimated dog activity as minor head or paw movement during sleep, as well as bouts of active dreaming (i.e., limb activity during rapid eye movement (REM) sleep), would have been coded as “active.” That being said, because our aim was to examine associations between human and dog activity, any dog movements occurring during sleep may still have influenced human activity and were therefore relevant to our analyses.

## Conclusions

This study contributes to the growing literature on the human-pet relationship, specifically co-sleeping between humans and their dogs. On one hand, we found no evidence for biological and demographic impacts on objective measures of human sleep or dog activity. On the other hand, we found that owners who had multiple dogs had worse self-reported sleep than those with only one dog. The mismatch between objective and subjective sleep quality has been widely reported in human sleep research [67]. Our time-series analysis offers a closer look at how human and dog activities co-vary through the night. As such, the question of “Who is disrupting whom?” remains inconclusive, as we found evidence of human activity preceding dog activity more often than dog activity preceded human activity; we emphasize that both parties impact the nighttime activity of one another—demonstrating our species’ close bond.

## Supporting information

S1 File Supporting tables and figure. Contains Tables A-C and Figure A.
(DOCX)

S2 File Canid sleep project questionnaire. (PDF)
