# Supplementary material for: Who is disrupting whom? Examining the nighttime activity patterns of humans and their dogs
Source: PLoS One. 2026 Oct 7;21(10):e0358453. doi: 10.1371/journal.pone.0358453 (PMC13645015; doi:10.1371/journal.pone.0358453)
Supplement: Supplementary file 1 [file pone.0358453.s002.pdf]

Canid Sleep Project Questionnaire

This questionnaire will be used to collect demographic information for our study. You can skip any question you do not wish to answer. Data will be de-identified for presentations and publication to guard participants' privacy. This first section should be completed with your information (owner), the second section should be completed with dog information, and the third section asks about co-sleep. Thank you for participating in this survey.

1. 1. Name (first, last)

   ____

2. 2. Date of birth

   Date/Month/Year

   ____

3. 3. Sex

   Check all that apply.

   - [ ] Female
   - [ ] Male
   - [ ] Other: ______________________________

4. 4. Gender

   ____

5. 5. Height

   State unit of measurement (cm, inches, feet)

   ____

6. 6. Weight

State unit of measurement (kilograms, pounds)

7. 7. State your dominant hand

The activity measuring device will be placed on your least dominant hand.

Mark only one oval.

- Left
- Right
- Ambidextrous

8. 8. Location

City & province/state & country

9. 9. List the number of pets that you have and indicate the species

10. 10. Amount of time spent with dog daily (estimate)

11. 11. Number of human co-sleepers

12. 12. Number of individuals in your household

Including yourself

13. 13. Are you the dog's primary caretaker?

Mark only one oval.

- Yes
- No
- Other:

14. 14. Number of animal co-sleepers

15. 15. How many times per day do you walk your dog and approximately how long are these walks (in minutes)?

If your dog engages in other physical activities (e.g., running, sledding), please provide this information as well as well how frequently they engage in these activities per week. Ex: Lily goes for daily walks that last 45 minutes, and Lily also practices sledding for 3 hours over the weekend.

16. 16. Please state your occupation and a rough estimate of how many hours you work weekly. Indicate whether you work from home or not.

17. 17. Optional: If you want to expand on any of your responses from Section 1, please do so here.

Dog information

Please fill out this section for your canid companion. If you have multiple dogs please number each for the following questions. (Example: name 1- Lily, 2- Rufus; sex 1- Female, 2- Male)

18. 1. Name

19. 2. Date of Birth

Date/Month/Year. If unknown provide year of birth or approximate age.

20. 3. Sex

Check all that apply.

- [ ] Female
- [ ] Male

21. 4. Breed

Please also indicate if your dog is pure bred or mixed.

____

22. 5. Weight

State unit of measurement

____

23. 6. Sleep location

Please include as much information as possible. Indicate whether the dog(s) sleeps indoors or outdoors. State sleeping area (e.g., floor, bed, crate, kennel).

____

24. 7. Number of human co-sleepers

How many people does your dog sleep with?

____

25. 8. Number of animal co-sleepers

____

26. 9. Approximate number of hours your dog spends alone daily

____

27. 10. Where does your dog spend most of their time during the daytime?

Mark only one oval.

- Indoors
- Outdoors
- Other: ____

28. 11. Diet

Please provide information regarding feeding schedules and whether or not your dog has food freely available to them. Indicate food types (e.g., dry, canned, human food). Example: Lily gets fed kibble 2x daily at 9 a.m. (1.5 cup) and 6 p.m. (2.5 cups)

29. 12. Reproductive status

Please indicate whether your dog is spayed/neutered.

30. 13. Please state any recent medical conditions

Could be allergies, injury, disease, or illness.

31. 14. Optional: If you want to expand on any of your responses from Section 2, please do so here.

____

____

____

____

____

Co-sleep

This section aims to gain insight about your general sleep patterns and how your dog influences your sleep.

32. 1. How many hours do you generally sleep per night?

Mark only one oval.

- Less than 6 hours
- 6-8 hours
- More than 8 hours

33. 2. How would you describe your sleep?

Mark only one oval.

	1	2	3	
Not				Too much

34. 3. Are you happy with your sleep?

Mark only one oval.

- ⬭ Yes
- ⬭ No
- ⬭ Other: ______

35. 4. How often does your dog sleep in your bed when you sleep?

Only fill out for the number of dogs you have. If you have multiple dogs, make sure the numbers correspond to the previous dog numbering.

Check all that apply.

	Always	Sometimes	Never
**Dog 1**	☐	☐	☐
**Dog 2**	☐	☐	☐
**Dog 3**	☐	☐	☐
**Dog 4**	☐	☐	☐

36. 5. Does your dog sleep in the same room that you sleep in?

Only fill out for the number of dogs you have. If you have multiple dogs, make sure the numbers correspond to the previous dog numbering.

Check all that apply.

	Always	Sometimes	Never
**Dog 1**	☐	☐	☐
**Dog 2**	☐	☐	☐
**Dog 3**	☐	☐	☐
**Dog 4**	☐	☐	☐

37. 6. Where does your dog sleep in relation to you for the majority of the night?

Can select all that apply or select other and provide more information. Only fill out for the number of dogs you have. If you have multiple dogs, make sure the numbers correspond to the previous dog numbering.

Check all that apply.

	Body contact	Less than 1m	1-3m	3-5m	More than 5m
**Dog 1**	☐	☐	☐	☐	☐
**Dog 2**	☐	☐	☐	☐	☐
**Dog 3**	☐	☐	☐	☐	☐
**Dog 4**	☐	☐	☐	☐	☐

38. 7. How do you feel your dog influences your sleep?

Mark only one oval.

	1	2	3	
For t	◯	◯	◯	For the worse

39. 8. How often do you wake/arouse from sleep at night?

Mark only one oval.

- ◯ Never
- ◯ Once
- ◯ Twice
- ◯ More than twice

40. 9. How often is your dog responsible for your arousal?

Mark only one oval.

- ◯ Never
- ◯ Once
- ◯ Twice
- ◯ More than twice
- ◯ Always

41. 10. Do you nap or sleep during the day? If yes, how many times during the day and please indicate the general duration.

____

42. 11. Does noise interrupt your sleep?

Mark only one oval.

- Yes
- No
- Other: ____

43. 12. Does your dog wake you up due to barking?

Mark only one oval.

- Yes
- No
- Other: ____

44. 13. Does your dog wake you up due to making noise from activity?

Mark only one oval.

- Yes
- No
- Other: ____

45. 14. Does having your dog sleep in the same home as you make you feel safer and more secure?

Mark only one oval.

- Yes
- No
- Other:

46. 15. State any patterns or trends you've recognized in your dog's sleep

Here you can include information about whether you notice movement while your dog sleeps, how easily they wake, whether they have habits before bed etc. If you have multiple dogs, make sure the numbers correspond to the previous dog numbering.

47. 16. During the study duration, did you have to remove the watch from yourself or the collar from your dog(s)? If so, please indicate the date and approximate time.

48. 17. During the study duration, were there any major changes or events that may have influenced your or your dog's activity patterns? If so, please indicate the date and approximate time.

Such as trip, moving, having visitors over, celebrations etc... Example: November 19th house party - Lily stayed up much later than usual (12 a.m. instead of her typical 9 p.m.)

49. 18. Optional: If you want to expand on any of your responses from Section 3, please do so here.

This content is neither created nor endorsed by Google.

Google Forms
