# Supplementary material for: Who is disrupting whom? Examining the nighttime activity patterns of humans and their dogs
Source: PLoS One. 2026 Oct 7;21(10):e0358453. doi: 10.1371/journal.pone.0358453 (PMC13645015; doi:10.1371/journal.pone.0358453)
Supplement: Supplementary file 2 [file pone.0358453.s001.docx]

**Supporting Information for “Who is disrupting whom? Examining the nighttime activity patterns of humans and their dogs”**

Ming Fei Li, Lavania Nagendran, Christopher Kiru, Lauren Schroeder, David R. Samson

**Table A.** Summary of demographic information for companion dogs. Dog ID and associated human ID is provided to note dyads included in this study. Sex, age and weight information is provided for each dog. Age (yr) and weight (kg) is described at the end of the data collection period. Number of nights of data collection for humans and dogs are included with the dog nights used for the cross-correlation analysis. Asterisks (*) next to ID notes dyads excluded or specific dates removed from analyses, see note below chart for more detailed reasoning. Table is modified from Nagendran et al. (2025; Supplementary Information).

| Human ID | Dog ID | Sex | Age (yr) | Weight (kg) | Num. of nights human/dogs |
| --- | --- | --- | --- | --- | --- |
| CAH016 | CAN061 | F | 6 | 21.77 | 33/31 |
| CAH017* | CAN062* | F | 4 | 24.94 | 26/24 |
| CAH018 | CAN063 | M | 9 | 28.12 | 34/32 |
| CAH019* | CAN064* | M | 2.8 | 38.10 | 27/23 |
| CAH020* | CAN065* | M | 3 | N/A | 0/0 |
| CAH020* | CAN066* | M | 2 | 23.35 | 23/20 |
| CAH021 | CAN067 | M | 9 | 27.21 | 41/37 |
| CAH021 | CAN068 | M | 10 | 24.94 | 41/37 |
| CAH022 | CAN069 | M | 3 | 30.84 | 35/33 |
| CAH023 | CAN070 | F | 2 | 19.95 | 34/31 |
| CAH023 | CAN071 | F | 2 | 18.14 | 34/31 |
| CAH024* | CAN072* | M | 4 | 29.48 | 32/23 |
| CAH025 | CAN073 | M | 11 | 19.95 | 35/32 |
| CAH025 | CAN074 | F | 6 | 15.78 | 35/32 |
| CAH026 | CAN075 | M | 2 | 31.75 | 33/31 |
| CAH027 | CAN076 | M | 4 | 37.64 | 36/34 |
| CAH028 | CAN077 | M | 2.5 | 61.24 | 34/32 |
| CAH028 | CAN078 | M | 8 | 24.94 | 34/30 |
| CAH029 | CAN079 | F | 8 | 24.94 | 40/37 |
| CAH030 | CAN080 | M | 6 | 39.91 | 34/32 |
| CAH031* | CAN081* | F | 4.5 | 32.65 | 31/26 |
| CAH032 | CAN082 | M | 7 | 34.01 | 34/30 |
| CAH033 | CAN083 | M | 6 | 44.45 | 34/32 |
| CAH033 | CAN084 | F | 3 | 34.01 | 34/32 |
| CAH033 | CAN085 | M | 5 | 72.57 | 34/32 |
| CAH033 | CAN086 | F | 5 | 36.28 | 34/32 |
| CAH034* | CAN087* | F | 6 | 31.75 | 29/27 |
| CAH035 | CAN088 | M | 5 | 31.75 | 34/32 |
| CAH035 | CAN089 | F | 3 | 16.78 | 34/32 |
| CAH035 | CAN090 | M | 3 | 27.21 | 34/32 |
| CAH035 | CAN091 | F | 7 | 20.41 | 34/32 |
| CAH036 | CAN092 | M | 2.5 | 29.02 | 34/32 |
| CAH036 | CAN093 | F | 3.5 | 24.94 | 34/32 |
| CAH036 | CAN094 | M | 8.5 | 33.11 | 34/32 |
| CAH037* | CAN095* | F | 2 | 20.41 | 0/0 |
| CAH038* | CAN096* | M | 3 | 24.94 | 31/29 |
| CAH039 | CAN097 | M | 3 | 25.85 | 34/31 |
| CAH040* | CAN098* | F | 2 | 24.94 | 19/16 |

*CAN095-CAH037 were excluded due to missing sensor while CAN065-CAH020 were excluded due to a majority of days missing data; CAN-098-CAH040 missing data November 12-15, 20, 27-29, December 1, 2; CAN062-CAH017 apart between November 5-14; CAN064-CAH019 apart between November 11-19; CAN066-CAH020 apart between November 8-18; CAN072-CAH024 missing data November 24, 26, 27, December 1, 3; CAN081-CAH031 missing data past December 1; CAN087-CAH034 apart November 29 - December 3; CAN096-CAH038 sensor removed December 4; CAN098-CAH040 missing data November 14-16.

**Table B.** Full model-averaged results from LMMs for human sleep measures (n = 777 observations). Reference categories for presence of other dogs, presence of human co-sleepers, and whether dogs sleep on the bed were set to “No” and for gender was “Woman.”

| Predictor | Estimate (SE) | p | 95% CI [low, high] |
| --- | --- | --- | --- |
| **Total Sleep Time (TST)** | | | |
| Presence of other dogs (Yes) | 0.003 (0.069) | 0.961 | [-0.271, 0.278] |
| Presence of human co-sleepers (Yes) | 0.011 (0.080) | 0.891 | [-0.320, 0.293] |
| Dogs sleep on bed (Sometimes) | -0.138 (0.162) | 0.396 | [-0.793, -0.120] |
| Dogs sleep on bed (Yes) | -0.087 (0.129) | 0.502 | [-0.590, -0.091] |
| Human age | -0.014 (0.089) | 0.871 | [-0.288, 0.259] |
| Human BMI | 0.069 (0.126) | 0.584 | [-0.160, 0.417] |
| Human gender (Man) | -0.012 (0.063) | 0.855 | [-0.285, 0.157] |
| **Sleep Efficiency (%)** | | | |
| Presence of other dogs (Yes) | -0.068 (0.120) | 0.569 | [-0.351, 0.215] |
| Presence of human co-sleepers (Yes) | -0.117 (0.147) | 0.424 | [-0.740, 0.071] |
| Dogs sleep on bed (Sometimes) | -0.138 (0.150) | 0.356 | [-0.739, 0.144] |
| Dogs sleep on bed (Yes) | -0.135 (0.149) | 0.364 | [-0.725, 0.161] |
| Human age | -0.199 (0.157) | 0.205 | [-0.769, 0.095] |
| Human BMI | 0.230 (0.170) | 0.178 | [-0.879, 0.670] |
| Human gender (Man) | 0.013 (0.098) | 0.894 | [-0.479, 0.319] |

**Table C.** Full model-averaged results from LMM for dog total nighttime activity (n = 1,093 observations). Reference categories for presence of other dogs and whether dogs sleep on the bed were set to “No,” for sex was “Female,” and for number of times fed was “>2.”

| Predictor | Estimate (SE) | p | 95% CI [low, high] |
| --- | --- | --- | --- |
| Presence of other dogs (Yes) | 0.050 (0.094) | 0.594 | [-0.134, 0.234] |
| Dogs sleep on bed (Sometimes) | -0.020 (0.065) | 0.764 | [-0.147, 0.108] |
| Dogs sleep on bed (Yes) | -0.040 (0.089) | 0.652 | [-0.215, 0.135] |
| Num. household members | -0.004 (0.038) | 0.921 | [-0.077, 0.070] |
| Num. feeding (1) | 0.004 (0.039) | 0.926 | [-0.074, 0.081] |
| Num. feeding (2) | -0.004 (0.039) | 0.915 | [-0.081, 0.073] |
| Dog age | 0.018 (0.062) | 0.765 | [-0.103, 0.140] |
| Dog weight | 0.007 (0.043) | 0.861 | [-0.076, 0.091] |
| Dog sex (Male) | 0.016 (0.059) | 0.788 | [-0.100, 0.132] |

**Figure A.** Boxplots illustrating Yule’s Y for lagged (-5 to +5) association between dog and human activity states across all nights. Dog ID is at the top of each plot. Peaks that are present are outlined by a box. Negative lag values represent human activity preceding dog activity, while positive values indicate human activity following dog activity and 0 suggesting synchronized activity within a 1-minute interval.
