# Supplementary material for: Who is disrupting whom? Examining the nighttime activity patterns of humans and their dogs
Source: PLoS One. 2026 Oct 7;21(10):e0358453. doi: 10.1371/journal.pone.0358453 (PMC13645015; doi:10.1371/journal.pone.0358453)
Supplement: Supplementary file 3 [file pone.0358453.s004.docx]

We thank the editor and two reviewers for providing constructive feedback. We have responded to each comment below, in red text.

Journal Requirements:

When submitting your revision, we need you to address these additional requirements.

1. Please ensure that your manuscript meets PLOS ONE's style requirements, including those for file naming. The PLOS ONE style templates can be found at

https://journals.plos.org/plosone/s/file?id=wjVg/PLOSOne_formatting_sample_main_body.pdf and

[https://journals.plos.org/plosone/s/file?id=ba62/PLOSOne_formatting_sample_title_authors_affiliations.pdf](https://nam11.safelinks.protection.outlook.com/?url=https%3A%2F%2Ftrack.editorialmanager.com%2FCL0%2Fhttps%3A%252F%252Fjournals.plos.org%252Fplosone%252Fs%252Ffile%253Fid%3Dba62%252FPLOSOne_formatting_sample_title_authors_affiliations.pdf%2F1%2F010f019f40f742dc-579ad12a-ce27-4273-a70a-75d2a06efaa5-000000%2FBoXI5nByLxXbAfaQlKECf9Q-NqxJ9QcMmSkjWYPYkcU%3D258&data=05%7C02%7Cmingfei.li%40duke.edu%7C88b7557d19fe4f0423b808dedccfb2de%7Ccb72c54e4a314d9eb14a1ea36dfac94c%7C0%7C0%7C639190982113247548%7CUnknown%7CTWFpbGZsb3d8eyJFbXB0eU1hcGkiOnRydWUsIlYiOiIwLjAuMDAwMCIsIlAiOiJXaW4zMiIsIkFOIjoiTWFpbCIsIldUIjoyfQ%3D%3D%7C0%7C%7C%7C&sdata=1EFSicJfptOWJcj9LN5r%2B3SYSOoQqG7VbuzQamg7zJw%3D&reserved=0)

2. We note that there is identifying data in the Supporting Information file "PONE_participant.info_human.sleep_psqi.xlsx". Due to the inclusion of these potentially identifying data, we have removed this file from your file inventory. Prior to sharing human research participant data, authors should consult with an ethics committee to ensure data are shared in accordance with participant consent and all applicable local laws.

Data sharing should never compromise participant privacy. It is therefore not appropriate to publicly share personally identifiable data on human research participants. The following are examples of data that should not be shared:

-Name, initials, physical address

-Ages more specific than whole numbers

-Internet protocol (IP) address

-Specific dates (birth dates, death dates, examination dates, etc.)

-Contact information such as phone number or email address

-Location data

-ID numbers that seem specific (long numbers, include initials, titled “Hospital ID”) rather than random (small numbers in numerical order)

Data that are not directly identifying may also be inappropriate to share, as in combination they can become identifying. For example, data collected from a small group of participants, vulnerable populations, or private groups should not be shared if they involve indirect identifiers (such as sex, ethnicity, location, etc.) that may risk the identification of study participants.

Additional guidance on preparing raw data for publication can be found in our Data Policy (https://journals.plos.org/plosone/s/data-availability#loc-human-research-participant-data-and-other-sensitive-data) and in the following article: http://www.bmj.com/content/340/bmj.c181.long.

Please remove or anonymize all personal information HumanID, age and gender, ensure that the data shared are in accordance with participant consent, and re-upload a fully anonymized data set. Please note that spreadsheet columns with personal information must be removed and not hidden as all hidden columns will appear in the published file.

The file "PONE_participant.info_human.sleep_psqi.xlsx" was removed from the Supporting Information since all relevant data files are now publicly available on OSF for data reproducibility. We have made sure that the data does not include identifying information (age is whole number, ID are randomly assigned).

3. Please include captions for your Supporting Information files at the end of your manuscript, and update any in-text citations to match accordingly. Please see our Supporting Information guidelines for more information: http://journals.plos.org/plosone/s/supporting-information.

The captions have been updated to reflect files uploaded and in-text citations are updates to match (line 629).

4. If the reviewer comments include a recommendation to cite specific previously published works, please review and evaluate these publications to determine whether they are relevant and should be cited. There is no requirement to cite these works unless the editor has indicated otherwise. 

Additional Editor Comments : 

Reviewer 1 Comments:

This was an interesting paper which I enjoyed reading. Most of my queries related to aspects of the methodology which I feel could be described better:

Title, short title and abstract: The titles and abstract suggest that all the dog-owner dyads were co-sleeping, yet only 16% of dogs always slept in the bed with 44% never sleeping in the bed and some not even in the same room. Perhaps the paper would be better titled something along the lines of the impact of co-sleeping versus not co-sleeping on humans and their dogs? Additionally, co-sleeping is not defined in the paper as used by the authors, and this could be a useful addition. I am assuming it means in the same bed, but perhaps this is not the definition intended?
We thank the reviewer for this suggestion. We have changed our title to: “*Who is disrupting whom? Examining the nighttime activity patterns of humans and their dogs.*” We now define the term “co-sleeping” as bedsharing in the abstract (line 14).

Line 74: Please specify the inclusion criteria here in terms of whether participants needed to co-sleep with their dogs to be enrolled. Additionally, where two adults co-slept, did both wear the monitor or only one? If the latter, how was the wearer chosen, and how was potential sleep disturbance caused by, and experienced by, the other human accounted for? Were any sample size calculations conducted eg was a specific ratio of co-sleepers versus not sought? Similarly, where there were multiple dogs, did they all wear monitors, and if not, how was the sentinel dog chosen?

- Participants did not need to co-sleep with dogs in order to participate and the ratio of individuals who co-sleep vs those that do not were not considered during the recruitment process

- We only collected activity data on one individual for each household (the person who reached out to participate and who identified as the primary caretaker).

- A questionnaire was used to collect further information about sleeping habits, arrangements, and possible disturbances in addition to collecting further information about each dog’s schedule. The questionnaire is now included in the Supporting Information.

-In the event that a household had multiple dogs that met the criteria (aged 2-12, was a husky or malamute, and was generally healthy) all dogs were included in the study. In the event that a dog of a multidog household could not participate we did not collect activity data for said dog but the questionnaire did account for this dog.

Changes in response to the above comments are reflected in lines 83-90.

Lines 79-80: Please provide further detail about which canine medical conditions led to exclusion, what level of diagnostic certainty was required (eg veterinary diagnosis versus owner self-report), and why these dogs were excluded. Were households excluded if any single dog had a medical condition, or were those households still included if another one of the dogs did not have a medical condition? Was dogs’ ability to hear considered? I am wondering if dogs sleeping outside the room would be disturbed when the humans moved around only if they could hear them.

We relied on owners’ self-reported canine medical conditions (line 79). We only excluded one dog from participating because they had impaired physical ability (e.g., three legs). One dog withdrew during the study duration due to the owner reporting they were sick—no further details were provided. To our knowledge, there were no existing medical conditions that would have affected dogs’ sleep-wake activity patterns (hearing). If one dog had to be excluded we still collected activity data from other dogs of the same household that met the criteria.

Line 85: Please provide further information about the actigraphy sensor used. For example, did it collect movement data in triaxial planes? This is mentioned in the discussion but would also be useful here.

The actigraphy sensor contains a tri-axial accelerometer that detects acceleration in three orthogonal (X, Y, Z) axes and uses the summation of these detected movements to generate an activity count per 1-minute epoch. We have included this information in the Methods section (lines 94-97).

Lines 89-90: Has this device subsequently been validated for detecting sleep/wake in dogs? This line alludes to the fact it has but I couldn’t see that information.

This device has not been validated for detecting sleep/wake in dogs, which we have pointed out. We have now added additional citations for studies that have used accelerometry to detect activity patterns in dogs, as well as other accelerometer devices that have been shown to predict behavioral sleep in dogs with high sensitivity and specificity when paired with behavioral observations (lines 103-109).

Line 103: Could the full survey be included in supplementary materials?

Full questionnaire is now included as supporting information (S2 File).

Line 119: What computer software was used for model building?
We added information on the software used for data analyses and the packages used for analyses (lines 191-196). We have also added a Data Availability statement (lines 198-199) and uploaded the files and code used on OSF (osf.io/7dt3c/).

Line 123-126: Were the data listed here captured per night or in summary for the period? Were any data collected about human noise-generating conditions rather than movements which might wake the dog eg snoring and sleep apnoea? Was BMI a proxy for this? Similarly, were the owners asked about whether their dog ever disturbed them at night in ways other than movement (eg snoring, barking or running during a dream)? Was human bed size taken into account as a potential factor for co-sleepers given the large size of these dogs? One might hypothesise that a super king size bed might lead to less disturbance than a small double? Finally, were human movements graded at all? It would seem logical that a person getting out of bed to go to the toilet would lead to more wakefulness in dogs anywhere in the house than the person remaining in bed whilst awake? If not, these could be additional limitations to discuss and/or factors to explore in future work. A daily diary for the humans to complete about the sleep locations of the dog(s) and any people, and any reasons for recalled nighttime waking causes could be useful to triangulate with the actigraphy data.

We thank the reviewer for pointing out these additional factors that might affect nighttime disturbances. We will address them one by one:

- This data was collected via a one-time questionnaire response collected at the end of the study period so it was not information collected per night. We have added this to the Materials and Methods section (lines 83-88; 118-119).

- We did not collect information on noise-generating conditions, and since we did not have video recordings or conduct behavioral observations, we do not know which behaviors may have generated the movements detected by the accelerometers. None of the human participants reported any medical conditions or sleep disorders (e.g., sleep apnea). We did not ask whether humans snored at night. We have added this to the Limitations section (lines 428-432).

- We did not note down the size of the bed. We have added this to the Discussion section for suggestions for future work (lines 401-402).

- We did ask owners whether their dogs disturb their sleep due to noise and barking and report this information in Table 1 and the Discussion, “Questionnaire responses suggest that humans did not feel like their dogs were interrupting their sleep. Out of 25 participants, the vast majority did not feel their sleep was interrupted by dog barking (88%) or noise caused by dog movements (84%). When we asked participants how often they wake up at night due to their dog(s), 14 participants reported zero times, nine participants said one or two times, and two participants said more than two times” (lines 364-368).

- Having a daily diary is an excellent suggestion for future studies—we have added this to our Discussion section (lines 399-401).

Lines 146-7: Please explain more clearly here what is meant by “number of feedings”. It is better explained in the discussion.

We have reworded to say the number of times fed per day (categorical: 1/2/ >2 times per day); lines 165-166.

Lines 160-162: Was all dog activity coded as wake? Could this have included bouts of active dreaming eg running in sleep?

Yes, all dog activity that was detected by the accelerometer (counts > 0) was coded as “active.” We have included this in the Limitations section as since we did not conduct behavioral observations of dogs, we could not verify the exact types of behavior that generated the movement captured by the accelerometer; thus, we may have overestimated the prevalence and duration of dog activity as slight movements of the head or paws during sleep, as well as bouts of active dreaming (i.e., limb activity during rapid eye movement (REM) sleep), would have been categorized as “active.” Changes are reflected in lines 432-438.

Line 198: For ease of reading, would it be possible to write in full the meanings of TST and SE here?

Yes, we now use the full terms (Total Sleep Time, Sleep Efficiency) instead of using acronyms throughout the Results (lines 218-219).

Limitations: See above for additional potential limitations.

Reviewer 2 Comments:

Thank you for the opportunity to review the manuscript entitled PONE-D-26-04064. The manuscript under consideration is both valid and of good quality in its investigation of sleep and nighttime activity in humans and dogs.
As there is currently contradictory evidence regarding the effect of dogs on humans' perceived sleep outcomes, and as studies exploring how co-sleeping affects the nighttime activity of dogs are limited, this investigation of 25 human–dog pairs using accelerometer and survey data provides important insights into the field. I found it particularly interesting that human activity often preceded dog activity. Overall, the manuscript is sound and well written, and I appreciate the thorough statistical analyses performed.

However, I have three concerns that I would like the authors to address (please also see the detailed comments below indicating the relevant row numbers):

1. Please provide your exact definition of co-sleeping. Does co-sleeping mean that the dog is in the same room, in the same bed, or something else? This definition will help readers better understand your analyses, results, and discussion.

In the Introduction, we now clearly define co-sleeping as bedsharing (line 37).

2. You state that co-sleeping had minimal effect on sleep duration or efficiency in humans and on nighttime activity in dogs. How was this assessed without using dogs sleeping in another room as a control group? Please clarify this in both the statistical methods and results sections.

In our analyses, we included whether “dogs slept on the same bed” as an independent variable in our models to evaluate co-sleeping and we assigned the control (reference) group as those who reported “no.” In other words, we were comparing dyads where the dogs sometimes/always co-slept on the same bed versus dogs that never co-slept on the bed (could be in the same room or a different room). We added this information to lines 139-144.

3. Has the CamNtech MotionWatch 8 actigraphy sensor been validated for detecting inactivity/activity in dogs? If so, please provide the relevant reference. If not, please describe how inactivity/activity in dogs was verified (e.g., by video recordings). How inactive is considered inactive? For example, are slight movements of the head or paws classified as inactivity? Please provide a clearer description of each activity state and discuss how this potential limitation of the device may have affected your results and conclusions.

This device has not been validated for detecting sleep/wake in dogs, which we have pointed out. We have now added additional citations for studies that have used accelerometry to detect activity patterns in dogs, as well as other accelerometer devices that have been shown to predict behavioral sleep in dogs with high sensitivity and specificity when paired with behavioral observations. Changes are reflected in lines 103-109.

We have added to the study Limitations that we did not conduct behavioral observations of dogs so we could not verify the exact types of behavior that generated the movement captured by the accelerometer; thus, we may have overestimated the duration of activity as slight movements of the head or paws would have been detected as activity. However, because our aim was to examine associations between human and dog activity, dog movements occurring during sleep may still have influenced human activity and were therefore relevant to our analyses. Changes are reflected in lines 432-438.

General comments on reference management:
When citing one or more references, readers would benefit from a brief indication of the most relevant or comparable findings from those references. For example, on row 247: “for studies comparing sleep in pet owners and non-owners, see [15,20,39]”. Please provide such guidance wherever applicable. In addition, please avoid mixing passive and active citation styles within the same sentence. See comments on rows 252 and 330.
We have written out the relevant findings and matched passive/active citation style within the same sentence (lines 277-280, 285, 362).

Abstract
Clear and concise, with relevant results that support the conclusions.

Introduction
The introduction is easy to understand and provides a good overview of the field. There are some minor language issues that could be improved; please see the comments below.
Typographical issue on row 33? The “e.g.” and the parentheses appear incorrect: (e.g., [10–13]).
Please remove the parentheses on row 39 and rewrite the sentence.

Parentheses have been removed.

I find that the frequent use of parentheses disrupts the flow of the text; please remove the parentheses on row 44 and rewrite the sentence.

Parentheses removed and sentence now reads “Co-sleeping, defined as bedsharing among humans and companion animals, is an extreme expression of multispecies entanglement” (line 37).

On row 45, there is another awkward use of parentheses (similar to row 33): (reviewed in [21]).

Parentheses have been removed and we have reworded that sentence (line 44-46).

The text describing the hypotheses is quite long and could benefit from being shortened and made more concise. Perhaps the text “…noise and movement. Considering the current inconclusive and contradictory evidence for the effect of pets on humans' perceived sleep outcomes” on rows 59–60 could be removed.

We have made the suggested changes (line 60).

Materials and Methods
The Materials and Methods section is missing information regarding the statistical software used, the packages included in the analyses, and the general significance level applied.

We have added this information to the Materials and Methods section (lines 191-196).

Please provide your exact definition of co-sleeping. Does it refer to having the dog in the same room, in the same bed, or something else? This definition will help readers better understand your analyses, results, and discussion.
We now clearly define co-sleeping as bedsharing in the Introduction (line 37), specified the reference (control) category in our data analyses (lines 141-142), and clarify co-sleeping in the Results (lines 205-206). We hope this clarifies any confusion about how we defined co-sleeping.

The statement on rows 88–89 is missing a reference: “has been widely used in studies of human sleep”.

We have added an appropriate reference (line 101-103).

Rows 89–90. Has the CamNtech MotionWatch 8 actigraphy sensor been validated for detecting inactivity/activity in dogs? If so, please provide the relevant reference. Otherwise, please describe how inactivity/activity in dogs was verified (e.g., by video recordings). How inactive is considered inactive? For example, are slight movements of the head or paws regarded as inactivity? Please provide a clearer description of each activity state.
Please see our response to comment 3 above (lines 103-109) and our Limitation section (lines 432-438).

Row 101. It is unclear whether “Lights Out” and “Got Up” were registered automatically by the device based on activity patterns or entered manually by the participants.

This was manually coded by one of the authors—we added more information about this to the Materials and Methods section (lines 113-116).

Rows 103–109. It is unclear whether participants completed the questionnaire once or multiple times. Were all questions answered at the end of the four-week registration period? Please clarify.
All questions were answered once at the end of the four-week study period. We have added the clarification (lines 83-85).

Row 112. Please clarify how missing data were defined. Was activity equal to zero for the humans, the dogs, or for both members of the pair? How was information regarding whether humans were with or apart from their dogs collected? (If the survey was completed only once and not daily.)
Missing data (non-wear time) was visually identified from looking at the raw data and defined as absence of activity (zero counts) for more than 30-minutes. The information about when humans were apart from the dogs was reported retroactively in the survey questionnaire at the end of the study or communicated to the research team during data collection period. Changes are reflected in lines 126-130.

Row 113. Could you please explain exactly what you mean by a dyad? I assume it refers to a human–dog pair whose data are analysed together, but I can´t see it stated. Please also clarify whether each human with more than one dog contributed more than one dyad, if applicable.
We defined dyad as “human-dog pair.” Humans with more than one dog contributed to more than one dyad. We have added this information (lines 130-132).

I appreciate the statistical descriptions of Analyses 1, 2, and 3. The response variables could perhaps be introduced at the beginning of each subsection, as they are currently somewhat difficult to locate. The statistical analyses are well described, but are the algorithms available? If possible, please consider providing also the full code as supplementary material.

We now introduce the response variable at the beginning of each Analysis section. All data and code used to process and analyze data are now publicly available on OSF (osf.io/7dt3c/); see Data Availability statement.

Results
Regarding the running text describing Table 1, is there perhaps too much overlap (double reporting) between the text and Table 1? The running text could potentially focus on the most important characteristics only.
We have condensed this section to focus on the most pertinent results (lines 202-209).

Perhaps the pronoun “we” does not belong in the table heading.
We have removed it (lines 212-214).

The Results section in general could perhaps benefit from subheadings corresponding to those used in the Discussion section.
We have added subheadings corresponding to those used in the Materials and Methods section: Analysis 1, 2, and 3.

It is not clearly described in the Results section that the non-co-sleepers (7/25) were used as a control group, if indeed they were. Was this group included as a reference category, intercept, or similar? I note from the legend of Table S2 that “sleep on bed” was coded as “no”, but what about dogs sleeping in another room?
We only looked at whether dogs slept in the same bed (“co-sleep”) as a variable in our models—the rationale being that if a dog slept in the same bed, then they were in the same room. We did not account for dogs that did not sleep on the same bed but slept in the same room versus dogs that slept in a different room. For dogs that did not sleep in the same room, they could have slept right outside the bedroom door or on another floor in the house—there was too much variability to adequately account for in our analyses. We feel that dog movement on the same bed versus not on the bed was the most likely variable to affect human sleep.

For the “co-sleeping” variable, the non-co-sleepers (11/25) were used as a control (reference) group. We have clarified this in the Materials and Methods section (lines 141-142) and have also reworded the Results to make it more clear (lines 205-209).

Discussion
What exactly is your definition of co-sleeping? Does it refer to having the dog in the same room or in the same bed? Define in M&M and discuss here.

We have now defined co-sleeping as bedsharing (line 37), and this can be with humans or companion animals (lines 205-209, 250).

Row 221. The term “cohabiting” has not been used previously. Please align the terminology with that used elsewhere in the manuscript.

We removed the term “cohabiting” (line 250).

Row 223. Is “they” the correct pronoun here? It is unclear what “they” refers to. In the same row, should it be “sleep quality” or “sleep efficiency”? These terms appear to be used interchangeably throughout the text.

We replaced “they” with “human co-sleepers” (line 252).

Row 233. Please add “objective” to the heading.

We have made the suggested change (line 263).

Rows 240–242. It is not entirely clear from the Results section that you included co-sleeping involving both humans and dogs; most of the text focuses on co-sleeping with the dog.

We have now included the percentage of participants that co-sleep with another person (lines 205-208) and this is also included in Table 1.

Rows 246–247. Regarding the statement, “As such, we could not assess whether the presence of dogs affected nighttime sleep in humans”, I wonder whether this could have been assessed using the non-co-sleepers as a comparison group. Please comment.

Our study did not find that co-sleeping had any significant association with sleep efficiency. To reiterate, our study did compare non-co-sleepers to co-sleepers however we did not include a sample of individuals that did not own dogs. The above statement is saying that we could not assess nighttime sleep in humans with versus without dogs. We have now made reference to two studies that compare sleep measures (objective and subjective) of non dog-owners and dog owners (lines 277-280).

Row 252. Please remove “by” before the reference. Also, please avoid mixing active and passive citation styles within the same sentence.

Removed.

Rows 287–288. Please remove the last sentence of this paragraph, as it does not appear to add any additional meaning.

Removed.

Rows 300–301. Are you referring to humans or dogs here? Please clarify.

We now clarified that this is referring to dogs (line 332).

Row 330. Please choose either an active or passive citation style; the sentence should not begin with a citation in parentheses.

We have fixed this (line 362).

Rows 355–360. Are you perhaps presenting new results here? I do not recognize these findings from the Results section. The information is very interesting, but it may need to be moved to the Results section and subsequently discussed here.

In our results, we now expand on findings from our time series analysis for correlated nighttime activities among co-sleeping human-dog dyads (sharing a bed, always, sometimes or never). Changes are reflected in lines 242-247.

Rows 374–376. The statement “Third, we set the MotionWatch 8 to triaxial ‘Mode 3,’ which was the setting recommended for detecting physical activity and movement along three axes” would benefit from being included in the Materials and Methods section.
We have added this information to the Materials and Methods section (lines 94-97).

In the limitations section, you should comment on the fact that the CamNtech MotionWatch 8 actigraphy sensor appear not to have been validated for detecting inactivity/activity in dogs. Please discuss how this limitation may have affected your results and conclusions.
We have expanded on this in the Limitations (lines 432-438).

In addition, the lack of a true control group should be discussed. I am not concerned that all participants were dog owners; this is an interesting and relevant study population in itself. Rather, I am a bit uncertain how the non-co-sleepers were handled in the analyses. You state that “Overall, co-sleeping had minimal effect on sleep duration or efficiency in humans or nighttime activity in dogs.” Is there a possible interaction between “in room” and “in bed”? Were the dogs that slept in another room always sleeping in another room? I may have missed something, but it is not entirely clear to me how this conclusion can be drawn from the model as currently described.
We changed this statement in our abstract from “minimal” to “no effect” to more accurately represent our results. It now reads: Overall, co-sleeping (i.e., bedsharing) had no effect on sleep duration or efficiency in humans or nighttime activity in dogs (line 14).

We agree and we have already pointed out in the Discussion that our study lacks a proper control group of humans who are non-pet-owners (line 274-277).

As we have responded to a previous comment: We only looked at whether dogs slept in the same bed (“co-sleep”) as a variable in our models—the rationale being that if a dog slept in the same bed, then they were in the same room. We did not account for dogs that did not sleep on the same bed but slept in the same room versus dogs that slept in a different room. For dogs that did not sleep in the same room, they could have slept right outside the bedroom door or on another floor in the house—there was too much variability to adequately account for in our analyses. We feel that dog movement on the same bed versus not on the bed was the most likely variable to affect human sleep. For this variable, the non-co-sleepers (11/25) were used as a control (reference) group. We have clarified this in the Materials and Methods section (lines 141-142) and have also reworded the Results to make it more clear (lines 205-209).

Apologies if we’re misunderstanding the comment. We do not see a way for the variables “in bed” and “in room” to interact because all dogs who sleep in the bed are already sleeping in the room; thus we cannot include both variables in our model. There is a possibility of only looking at dyads who sleep in the same room (n = 12; 1 dyad excluded for missing data) and then further dividing them into dogs that always (n = 3), sometimes (n = 7), or never (n = 2) sleep in the bed, but unfortunately, we do not have a large enough sample size per group to fit our statistical models.

Conclusions
Perhaps the following text could be removed:
“…and subjective sleep may be a stronger predictor of health outcomes [56]. The advent and popularization of wearable technologies have allowed researchers to tap into less-explored spatiotemporal dimensions of human–pet cohabitation.”

These sections have been removed.

I would instead suggest moving the following text to the Discussion section:
“Investigations into human and dog activity and sleep have been and will continue to be subject to the inevitable influence of cultural factors and individual variations that are difficult to capture in statistical models [57,58].”
Regarding the statement “More nuanced approaches are clearly needed”, please provide more specific directions for future research based on your main findings.

These statements have been moved to the Discussion preceding our Limitations (404-407).

Thank you for a well performed study and well written manuscript. I would be happy to review the revised manuscript
