# Supplementary material for: Who is disrupting whom? Examining the nighttime activity patterns of humans and their dogs
Source: PLoS One. 2026 Oct 7;21(10):e0358453. doi: 10.1371/journal.pone.0358453 (PMC13645015; doi:10.1371/journal.pone.0358453)
Supplement: Supplementary file 4 [file pone.0358453.s005.docx]

We thank the editor and reviewers for their feedback on our manuscript. We have responded to reviewer comments below, in red text.

Reviewer #1: Many thanks for your detailed response to our comments. The manuscript is much clearer as a result. From me, two very minor comments remain:

Lines 242-274: might benefit from some interpretation of what the lag times mean, as I think many people will be unfamiliar with this analysis. I appreciate this is included in the methods, but a little reminder here might do no harm!

Thanks for this suggestion. We have added more explanation to make this section clearer for readers (lines 238-243).

Limitations: Given that the single survey was completed at the end of a four week period, this is likely to have introduced some recall bias which should be mentioned as a limitation - perhaps where you talk about use of a diary as an alternative?
We have added this to the limitations (lines 405-407).
