# Supplementary material for: Who is disrupting whom? Examining the nighttime activity patterns of humans and their dogs
Source: PLoS One. 2026 Oct 7;21(10):e0358453. doi: 10.1371/journal.pone.0358453 (PMC13645015; doi:10.1371/journal.pone.0358453)
Supplement: Peer review history

# Peer review: Decision Letter 0

**DOI:** 10.1371/journal.pone.0358453.r001

**Authors:** Stanley Chinedu Eneh

8 Jul 2026

PONE-D-26-04064

Who is disrupting whom? The impact of co-sleeping on humans and their dogs

PLOS One?

Dear Dr. Li,

Thank you for submitting your manuscript to PLOS ONE. After careful consideration, we feel that it has merit but does not fully meet PLOS ONE’s publication criteria as it currently stands. Therefore, we invite you to submit a revised version of the manuscript that addresses the points raised during the review process. Kindly find the reviewer's Comments below.

Please submit your revised manuscript by Aug 22 2026 11:59PM. If you will need more time than this to complete your revisions, please reply to this message or contact the journal office at plosone@plos.org. When you're ready to submit your revision, log on to https://www.editorialmanager.com/pone/ and select the 'Submissions Needing Revision' folder to locate your manuscript file.

- A letter that responds to each point raised by the academic editor and reviewer(s). You should upload this letter as a separate file labeled 'Response to Reviewers'.
- A marked-up copy of your manuscript that highlights changes made to the original version. You should upload this as a separate file labeled 'Revised Manuscript with Track Changes'.
- An unmarked version of your revised paper without tracked changes. You should upload this as a separate file labeled 'Manuscript'.

If you would like to make changes to your financial disclosure, please include your updated statement in your cover letter. Guidelines for resubmitting your figure files are available below the reviewer comments at the end of this letter.

If applicable, we recommend that you deposit your laboratory protocols in protocols.io to enhance the reproducibility of your results. Protocols.io assigns your protocol its own identifier (DOI) so that it can be cited independently in the future. For instructions see: https://journals.plos.org/plosone/s/submission-guidelines#loc-laboratory-protocols. Additionally, PLOS ONE offers an option for publishing peer-reviewed Lab Protocol articles, which describe protocols hosted on protocols.io. Read more information on sharing protocols at https://plos.org/protocols?utm_medium=editorial-email&utm_source=authorletters&utm_campaign=protocols.

As the corresponding author, your ORCID iD is verified in the submission system and will appear in the published article. PLOS supports the use of ORCID, and we encourage all coauthors to register for an ORCID iD and use it as well. Please encourage your coauthors to verify their ORCID iD within the submission system before final acceptance, as unverified ORCID iDs will not appear in the published article. *Only*  the individual author can complete the verification step; PLOS staff *cannot*  verify ORCID iDs on behalf of authors.

We look forward to receiving your revised manuscript.

Kind regards,

Stanley Chinedu Eneh

Academic Editor

PLOS One

Journal Requirements:

When submitting your revision, we need you to address these additional requirements.

1. Please ensure that your manuscript meets PLOS ONE's style requirements, including those for file naming. The PLOS ONE style templates can be found at

https://journals.plos.org/plosone/s/file?id=wjVg/PLOSOne_formatting_sample_main_body.pdf and

https://journals.plos.org/plosone/s/file?id=ba62/PLOSOne_formatting_sample_title_authors_affiliations.pdf

2. We note that there is identifying data in the Supporting Information file "PONE_participant.info_human.sleep_psqi.xlsx". Due to the inclusion of these potentially identifying data, we have removed this file from your file inventory. Prior to sharing human research participant data, authors should consult with an ethics committee to ensure data are shared in accordance with participant consent and all applicable local laws.

Data sharing should never compromise participant privacy. It is therefore not appropriate to publicly share personally identifiable data on human research participants. The following are examples of data that should not be shared:

-Name, initials, physical address

-Ages more specific than whole numbers

-Internet protocol (IP) address

-Specific dates (birth dates, death dates, examination dates, etc.)

-Contact information such as phone number or email address

-Location data

-ID numbers that seem specific (long numbers, include initials, titled “Hospital ID”) rather than random (small numbers in numerical order)

Data that are not directly identifying may also be inappropriate to share, as in combination they can become identifying. For example, data collected from a small group of participants, vulnerable populations, or private groups should not be shared if they involve indirect identifiers (such as sex, ethnicity, location, etc.) that may risk the identification of study participants.

Additional guidance on preparing raw data for publication can be found in our Data Policy (https://journals.plos.org/plosone/s/data-availability#loc-human-research-participant-data-and-other-sensitive-data) and in the following article: http://www.bmj.com/content/340/bmj.c181.long.

Please remove or anonymize all personal information HumanID, age and gender, ensure that the data shared are in accordance with participant consent, and re-upload a fully anonymized data set. Please note that spreadsheet columns with personal information must be removed and not hidden as all hidden columns will appear in the published file.

3. Please include captions for your Supporting Information files at the end of your manuscript, and update any in-text citations to match accordingly. Please see our Supporting Information guidelines for more information: http://journals.plos.org/plosone/s/supporting-information.

4. If the reviewer comments include a recommendation to cite specific previously published works, please review and evaluate these publications to determine whether they are relevant and should be cited. There is no requirement to cite these works unless the editor has indicated otherwise.

Additional Editor Comments :

Reviewer 1 Comments:

This was an interesting paper which I enjoyed reading. Most of my queries related to aspects of the methodology which I feel could be described better:

Title, short title and abstract: The titles and abstract suggest that all the dog-owner dyads were co-sleeping, yet only 16% of dogs always slept in the bed with 44% never sleeping in the bed and some not even in the same room. Perhaps the paper would be better titled something along the lines of the impact of co-sleeping versus not co-sleeping on humans and their dogs? Additionally, co-sleeping is not defined in the paper as used by the authors, and this could be a useful addition. I am assuming it means in the same bed, but perhaps this is not the definition intended?

Line 74: Please specify the inclusion criteria here in terms of whether participants needed to co-sleep with their dogs to be enrolled. Additionally, where two adults co-slept, did both wear the monitor or only one? If the latter, how was the wearer chosen, and how was potential sleep disturbance caused by, and experienced by, the other human accounted for? Were any sample size calculations conducted eg was a specific ratio of co-sleepers versus not sought? Similarly, where there were multiple dogs, did they all wear monitors, and if not, how was the sentinel dog chosen?

Lines 79-80: Please provide further detail about which canine medical conditions led to exclusion, what level of diagnostic certainty was required (eg veterinary diagnosis versus owner self-report), and why these dogs were excluded. Were households excluded if any single dog had a medical condition, or were those households still included if another one of the dogs did not have a medical condition? Was dogs’ ability to hear considered? I am wondering if dogs sleeping outside the room would be disturbed when the humans moved around only if they could hear them.

Line 85: Please provide further information about the actigraphy sensor used. For example, did it collect movement data in triaxial planes? This is mentioned in the discussion but would also be useful here.

Lines 89-90: Has this device subsequently been validated for detecting sleep/wake in dogs? This line alludes to the fact it has but I couldn’t see that information.

Line 103: Could the full survey be included in supplementary materials?

Line 119: What computer software was used for model building?

Line 123-126: Were the data listed here captured per night or in summary for the period? Were any data collected about human noise-generating conditions rather than movements which might wake the dog eg snoring and sleep apnoea? Was BMI a proxy for this? Similarly, were the owners asked about whether their dog ever disturbed them at night in ways other than movement (eg snoring, barking or running during a dream)? Was human bed size taken into account as a potential factor for co-sleepers given the large size of these dogs? One might hypothesise that a super king size bed might lead to less disturbance than a small double? Finally, were human movements graded at all? It would seem logical that a person getting out of bed to go to the toilet would lead to more wakefulness in dogs anywhere in the house than the person remaining in bed whilst awake? If not, these could be additional limitations to discuss and/or factors to explore in future work. A daily diary for the humans to complete about the sleep locations of the dog(s) and any people, and any reasons for recalled nighttime waking causes could be useful to triangulate with the actigraphy data.

Lines 146-7: Please explain more clearly here what is meant by “number of feedings”. It is better explained in the discussion.

Lines 160-162: Was all dog activity coded as wake? Could this have included bouts of active dreaming eg running in sleep?

Line 198: For ease of reading, would it be possible to write in full the meanings of TST and SE here?

Limitations: See above for additional potential limitations.

Reviewer 2 Comments:

Thank you for the opportunity to review the manuscript entitled PONE-D-26-04064. The manuscript under consideration is both valid and of good quality in its investigation of sleep and nighttime activity in humans and dogs.

As there is currently contradictory evidence regarding the effect of dogs on humans' perceived sleep outcomes, and as studies exploring how co-sleeping affects the nighttime activity of dogs are limited, this investigation of 25 human–dog pairs using accelerometer and survey data provides important insights into the field. I found it particularly interesting that human activity often preceded dog activity. Overall, the manuscript is sound and well written, and I appreciate the thorough statistical analyses performed.

However, I have three concerns that I would like the authors to address (please also see the detailed comments below indicating the relevant row numbers):

1. Please provide your exact definition of co-sleeping. Does co-sleeping mean that the dog is in the same room, in the same bed, or something else? This definition will help readers better understand your analyses, results, and discussion.

2. You state that co-sleeping had minimal effect on sleep duration or efficiency in humans and on nighttime activity in dogs. How was this assessed without using dogs sleeping in another room as a control group? Please clarify this in both the statistical methods and results sections.

3. Has the CamNtech MotionWatch 8 actigraphy sensor been validated for detecting inactivity/activity in dogs? If so, please provide the relevant reference. If not, please describe how inactivity/activity in dogs was verified (e.g., by video recordings). How inactive is considered inactive? For example, are slight movements of the head or paws classified as inactivity? Please provide a clearer description of each activity state and discuss how this potential limitation of the device may have affected your results and conclusions.

General comments on reference management:

When citing one or more references, readers would benefit from a brief indication of the most relevant or comparable findings from those references. For example, on row 247: “for studies comparing sleep in pet owners and non-owners, see [15,20,39]”. Please provide such guidance wherever applicable. In addition, please avoid mixing passive and active citation styles within the same sentence. See comments on rows 252 and 330.

Abstract

Clear and concise, with relevant results that support the conclusions.

Introduction

The introduction is easy to understand and provides a good overview of the field. There are some minor language issues that could be improved; please see the comments below.

Typographical issue on row 33? The “e.g.” and the parentheses appear incorrect: (e.g., [10–13]).

Please remove the parentheses on row 39 and rewrite the sentence.

I find that the frequent use of parentheses disrupts the flow of the text; please remove the parentheses on row 44 and rewrite the sentence.

On row 45, there is another awkward use of parentheses (similar to row 33): (reviewed in [21]).

The text describing the hypotheses is quite long and could benefit from being shortened and made more concise. Perhaps the text “…noise and movement. Considering the current inconclusive and contradictory evidence for the effect of pets on humans' perceived sleep outcomes” on rows 59–60 could be removed.

Materials and Methods

The Materials and Methods section is missing information regarding the statistical software used, the packages included in the analyses, and the general significance level applied.

Please provide your exact definition of co-sleeping. Does it refer to having the dog in the same room, in the same bed, or something else? This definition will help readers better understand your analyses, results, and discussion.

The statement on rows 88–89 is missing a reference: “has been widely used in studies of human sleep”.

Rows 89–90. Has the CamNtech MotionWatch 8 actigraphy sensor been validated for detecting inactivity/activity in dogs? If so, please provide the relevant reference. Otherwise, please describe how inactivity/activity in dogs was verified (e.g., by video recordings). How inactive is considered inactive? For example, are slight movements of the head or paws regarded as inactivity? Please provide a clearer description of each activity state.

Row 101. It is unclear whether “Lights Out” and “Got Up” were registered automatically by the device based on activity patterns or entered manually by the participants.

Rows 103–109. It is unclear whether participants completed the questionnaire once or multiple times. Were all questions answered at the end of the four-week registration period? Please clarify.

Row 112. Please clarify how missing data were defined. Was activity equal to zero for the humans, the dogs, or for both members of the pair? How was information regarding whether humans were with or apart from their dogs collected? (If the survey was completed only once and not daily.)

Row 113. Could you please explain exactly what you mean by a dyad? I assume it refers to a human–dog pair whose data are analysed together, but I can´t see it stated. Please also clarify whether each human with more than one dog contributed more than one dyad, if applicable.

I appreciate the statistical descriptions of Analyses 1, 2, and 3. The response variables could perhaps be introduced at the beginning of each subsection, as they are currently somewhat difficult to locate. The statistical analyses are well described, but are the algorithms available? If possible, please consider providing also the full code as supplementary material.

Results

Regarding the running text describing Table 1, is there perhaps too much overlap (double reporting) between the text and Table 1? The running text could potentially focus on the most important characteristics only.

Perhaps the pronoun “we” does not belong in the table heading.

The Results section in general could perhaps benefit from subheadings corresponding to those used in the Discussion section.

It is not clearly described in the Results section that the non-co-sleepers (7/25) were used as a control group, if indeed they were. Was this group included as a reference category, intercept, or similar? I note from the legend of Table S2 that “sleep on bed” was coded as “no”, but what about dogs sleeping in another room?

Discussion

What exactly is your definition of co-sleeping? Does it refer to having the dog in the same room or in the same bed? Define in M&M and discuss here.

Row 221. The term “cohabiting” has not been used previously. Please align the terminology with that used elsewhere in the manuscript.

Row 223. Is “they” the correct pronoun here? It is unclear what “they” refers to. In the same row, should it be “sleep quality” or “sleep efficiency”? These terms appear to be used interchangeably throughout the text.

Row 233. Please add “objective” to the heading.

Rows 240–242. It is not entirely clear from the Results section that you included co-sleeping involving both humans and dogs; most of the text focuses on co-sleeping with the dog.

Rows 246–247. Regarding the statement, “As such, we could not assess whether the presence of dogs affected nighttime sleep in humans”, I wonder whether this could have been assessed using the non-co-sleepers as a comparison group. Please comment.

Row 252. Please remove “by” before the reference. Also, please avoid mixing active and passive citation styles within the same sentence.

Rows 287–288. Please remove the last sentence of this paragraph, as it does not appear to add any additional meaning.

Rows 300–301. Are you referring to humans or dogs here? Please clarify.

Row 330. Please choose either an active or passive citation style; the sentence should not begin with a citation in parentheses.

Rows 355–360. Are you perhaps presenting new results here? I do not recognize these findings from the Results section. The information is very interesting, but it may need to be moved to the Results section and subsequently discussed here.

Rows 374–376. The statement “Third, we set the MotionWatch 8 to triaxial ‘Mode 3,’ which was the setting recommended for detecting physical activity and movement along three axes” would benefit from being included in the Materials and Methods section.

In the limitations section, you should comment on the fact that the CamNtech MotionWatch 8 actigraphy sensor appear not to have been validated for detecting inactivity/activity in dogs. Please discuss how this limitation may have affected your results and conclusions.

In addition, the lack of a true control group should be discussed. I am not concerned that all participants were dog owners; this is an interesting and relevant study population in itself. Rather, I am a bit uncertain how the non-co-sleepers were handled in the analyses. You state that “Overall, co-sleeping had minimal effect on sleep duration or efficiency in humans or nighttime activity in dogs.” Is there a possible interaction between “in room” and “in bed”? Were the dogs that slept in another room always sleeping in another room? I may have missed something, but it is not entirely clear to me how this conclusion can be drawn from the model as currently described.

Conclusions

Perhaps the following text could be removed:

“…and subjective sleep may be a stronger predictor of health outcomes [56]. The advent and popularization of wearable technologies have allowed researchers to tap into less-explored spatiotemporal dimensions of human–pet cohabitation.”

I would instead suggest moving the following text to the Discussion section:

“Investigations into human and dog activity and sleep have been and will continue to be subject to the inevitable influence of cultural factors and individual variations that are difficult to capture in statistical models [57,58].”

Regarding the statement “More nuanced approaches are clearly needed”, please provide more specific directions for future research based on your main findings.

Thank you for a well performed study and well written manuscript. I would be happy to review the revised manuscript

[Note: HTML markup is below. Please do not edit.]

Reviewers' comments:

Reviewer's Responses to Questions

**Comments to the Author**

1. Is the manuscript technically sound, and do the data support the conclusions?

Reviewer #1: Yes

Reviewer #2: Partly

**********

2. Has the statistical analysis been performed appropriately and rigorously?

Reviewer #1: I Don't Know

Reviewer #2: Yes

**********

3. Have the authors made all data underlying the findings in their manuscript fully available?

The PLOS Data policy

Reviewer #1: Yes

Reviewer #2: Yes

**********

4. Is the manuscript presented in an intelligible fashion and written in standard English?

Reviewer #1: Yes

Reviewer #2: Yes

**********

Reviewer #1: This was an interesting paper which I enjoyed reading. Most of my queries related to aspects of the methodology which I feel could be described better:

Title, short title and abstract: The titles and abstract suggest that all the dog-owner dyads were co-sleeping, yet only 16% of dogs always slept in the bed with 44% never sleeping in the bed and some not even in the same room. Perhaps the paper would be better titled something along the lines of the impact of co-sleeping versus not co-sleeping on humans and their dogs? Additionally, co-sleeping is not defined in the paper as used by the authors, and this could be a useful addition. I am assuming it means in the same bed, but perhaps this is not the definition intended?

Line 74: Please specify the inclusion criteria here in terms of whether participants needed to co-sleep with their dogs to be enrolled. Additionally, where two adults co-slept, did both wear the monitor or only one? If the latter, how was the wearer chosen, and how was potential sleep disturbance caused by, and experienced by, the other human accounted for? Were any sample size calculations conducted eg was a specific ratio of co-sleepers versus not sought? Similarly, where there were multiple dogs, did they all wear monitors, and if not, how was the sentinel dog chosen?

Lines 79-80: Please provide further detail about which canine medical conditions led to exclusion, what level of diagnostic certainty was required (eg veterinary diagnosis versus owner self-report), and why these dogs were excluded. Were households excluded if any single dog had a medical condition, or were those households still included if another one of the dogs did not have a medical condition? Was dogs’ ability to hear considered? I am wondering if dogs sleeping outside the room would be disturbed when the humans moved around only if they could hear them.

Line 85: Please provide further information about the actigraphy sensor used. For example, did it collect movement data in triaxial planes? This is mentioned in the discussion but would also be useful here.

Lines 89-90: Has this device subsequently been validated for detecting sleep/wake in dogs? This line alludes to the fact it has but I couldn’t see that information.

Line 103: Could the full survey be included in supplementary materials?

Line 119: What computer software was used for model building?

Line 123-126: Were the data listed here captured per night or in summary for the period? Were any data collected about human noise-generating conditions rather than movements which might wake the dog eg snoring and sleep apnoea? Was BMI a proxy for this? Similarly, were the owners asked about whether their dog ever disturbed them at night in ways other than movement (eg snoring, barking or running during a dream)? Was human bed size taken into account as a potential factor for co-sleepers given the large size of these dogs? One might hypothesise that a super king size bed might lead to less disturbance than a small double? Finally, were human movements graded at all? It would seem logical that a person getting out of bed to go to the toilet would lead to more wakefulness in dogs anywhere in the house than the person remaining in bed whilst awake? If not, these could be additional limitations to discuss and/or factors to explore in future work. A daily diary for the humans to complete about the sleep locations of the dog(s) and any people, and any reasons for recalled nighttime waking causes could be useful to triangulate with the actigraphy data.

Lines 146-7: Please explain more clearly here what is meant by “number of feedings”. It is better explained in the discussion.

Lines 160-162: Was all dog activity coded as wake? Could this have included bouts of active dreaming eg running in sleep?

Line 198: For ease of reading, would it be possible to write in full the meanings of TST and SE here?

Limitations: See above for additional potential limitations.

Reviewer #2: PLOS One: Agreement to Review PONE-D-26-04064 - [EMID:dd8ee70e4ffe05b0]

Dear Editor of PLOS One and Authors,

Thank you for the opportunity to review the manuscript entitled PONE-D-26-04064. The manuscript under consideration is both valid and of good quality in its investigation of sleep and nighttime activity in humans and dogs.

As there is currently contradictory evidence regarding the effect of dogs on humans' perceived sleep outcomes, and as studies exploring how co-sleeping affects the nighttime activity of dogs are limited, this investigation of 25 human–dog pairs using accelerometer and survey data provides important insights into the field. I found it particularly interesting that human activity often preceded dog activity. Overall, the manuscript is sound and well written, and I appreciate the thorough statistical analyses performed.

However, I have three concerns that I would like the authors to address (please also see the detailed comments below indicating the relevant row numbers):

1. Please provide your exact definition of co-sleeping. Does co-sleeping mean that the dog is in the same room, in the same bed, or something else? This definition will help readers better understand your analyses, results, and discussion.

2. You state that co-sleeping had minimal effect on sleep duration or efficiency in humans and on nighttime activity in dogs. How was this assessed without using dogs sleeping in another room as a control group? Please clarify this in both the statistical methods and results sections.

3. Has the CamNtech MotionWatch 8 actigraphy sensor been validated for detecting inactivity/activity in dogs? If so, please provide the relevant reference. If not, please describe how inactivity/activity in dogs was verified (e.g., by video recordings). How inactive is considered inactive? For example, are slight movements of the head or paws classified as inactivity? Please provide a clearer description of each activity state and discuss how this potential limitation of the device may have affected your results and conclusions.

General comments on reference management:

When citing one or more references, readers would benefit from a brief indication of the most relevant or comparable findings from those references. For example, on row 247: “for studies comparing sleep in pet owners and non-owners, see [15,20,39]”. Please provide such guidance wherever applicable. In addition, please avoid mixing passive and active citation styles within the same sentence. See comments on rows 252 and 330.

Abstract

Clear and concise, with relevant results that support the conclusions.

Introduction

The introduction is easy to understand and provides a good overview of the field. There are some minor language issues that could be improved; please see the comments below.

Typographical issue on row 33? The “e.g.” and the parentheses appear incorrect: (e.g., [10–13]).

Please remove the parentheses on row 39 and rewrite the sentence.

I find that the frequent use of parentheses disrupts the flow of the text; please remove the parentheses on row 44 and rewrite the sentence.

On row 45, there is another awkward use of parentheses (similar to row 33): (reviewed in [21]).

The text describing the hypotheses is quite long and could benefit from being shortened and made more concise. Perhaps the text “…noise and movement. Considering the current inconclusive and contradictory evidence for the effect of pets on humans' perceived sleep outcomes” on rows 59–60 could be removed.

Materials and Methods

The Materials and Methods section is missing information regarding the statistical software used, the packages included in the analyses, and the general significance level applied.

Please provide your exact definition of co-sleeping. Does it refer to having the dog in the same room, in the same bed, or something else? This definition will help readers better understand your analyses, results, and discussion.

The statement on rows 88–89 is missing a reference: “has been widely used in studies of human sleep”.

Rows 89–90. Has the CamNtech MotionWatch 8 actigraphy sensor been validated for detecting inactivity/activity in dogs? If so, please provide the relevant reference. Otherwise, please describe how inactivity/activity in dogs was verified (e.g., by video recordings). How inactive is considered inactive? For example, are slight movements of the head or paws regarded as inactivity? Please provide a clearer description of each activity state.

Row 101. It is unclear whether “Lights Out” and “Got Up” were registered automatically by the device based on activity patterns or entered manually by the participants.

Rows 103–109. It is unclear whether participants completed the questionnaire once or multiple times. Were all questions answered at the end of the four-week registration period? Please clarify.

Row 112. Please clarify how missing data were defined. Was activity equal to zero for the humans, the dogs, or for both members of the pair? How was information regarding whether humans were with or apart from their dogs collected? (If the survey was completed only once and not daily.)

Row 113. Could you please explain exactly what you mean by a dyad? I assume it refers to a human–dog pair whose data are analysed together, but I can´t see it stated. Please also clarify whether each human with more than one dog contributed more than one dyad, if applicable.

I appreciate the statistical descriptions of Analyses 1, 2, and 3. The response variables could perhaps be introduced at the beginning of each subsection, as they are currently somewhat difficult to locate. The statistical analyses are well described, but are the algorithms available? If possible, please consider providing also the full code as supplementary material.

Results

Regarding the running text describing Table 1, is there perhaps too much overlap (double reporting) between the text and Table 1? The running text could potentially focus on the most important characteristics only.

Perhaps the pronoun “we” does not belong in the table heading.

The Results section in general could perhaps benefit from subheadings corresponding to those used in the Discussion section.

It is not clearly described in the Results section that the non-co-sleepers (7/25) were used as a control group, if indeed they were. Was this group included as a reference category, intercept, or similar? I note from the legend of Table S2 that “sleep on bed” was coded as “no”, but what about dogs sleeping in another room?

Discussion

What exactly is your definition of co-sleeping? Does it refer to having the dog in the same room or in the same bed? Define in M&M and discuss here.

Row 221. The term “cohabiting” has not been used previously. Please align the terminology with that used elsewhere in the manuscript.

Row 223. Is “they” the correct pronoun here? It is unclear what “they” refers to. In the same row, should it be “sleep quality” or “sleep efficiency”? These terms appear to be used interchangeably throughout the text.

Row 233. Please add “objective” to the heading.

Rows 240–242. It is not entirely clear from the Results section that you included co-sleeping involving both humans and dogs; most of the text focuses on co-sleeping with the dog.

Rows 246–247. Regarding the statement, “As such, we could not assess whether the presence of dogs affected nighttime sleep in humans”, I wonder whether this could have been assessed using the non-co-sleepers as a comparison group. Please comment.

Row 252. Please remove “by” before the reference. Also, please avoid mixing active and passive citation styles within the same sentence.

Rows 287–288. Please remove the last sentence of this paragraph, as it does not appear to add any additional meaning.

Rows 300–301. Are you referring to humans or dogs here? Please clarify.

Row 330. Please choose either an active or passive citation style; the sentence should not begin with a citation in parentheses.

Rows 355–360. Are you perhaps presenting new results here? I do not recognize these findings from the Results section. The information is very interesting, but it may need to be moved to the Results section and subsequently discussed here.

Rows 374–376. The statement “Third, we set the MotionWatch 8 to triaxial ‘Mode 3,’ which was the setting recommended for detecting physical activity and movement along three axes” would benefit from being included in the Materials and Methods section.

In the limitations section, you should comment on the fact that the CamNtech MotionWatch 8 actigraphy sensor appear not to have been validated for detecting inactivity/activity in dogs. Please discuss how this limitation may have affected your results and conclusions.

In addition, the lack of a true control group should be discussed. I am not concerned that all participants were dog owners; this is an interesting and relevant study population in itself. Rather, I am a bit uncertain how the non-co-sleepers were handled in the analyses. You state that “Overall, co-sleeping had minimal effect on sleep duration or efficiency in humans or nighttime activity in dogs.” Is there a possible interaction between “in room” and “in bed”? Were the dogs that slept in another room always sleeping in another room? I may have missed something, but it is not entirely clear to me how this conclusion can be drawn from the model as currently described.

Conclusions

Perhaps the following text could be removed:

“…and subjective sleep may be a stronger predictor of health outcomes [56]. The advent and popularization of wearable technologies have allowed researchers to tap into less-explored spatiotemporal dimensions of human–pet cohabitation.”

I would instead suggest moving the following text to the Discussion section:

“Investigations into human and dog activity and sleep have been and will continue to be subject to the inevitable influence of cultural factors and individual variations that are difficult to capture in statistical models [57,58].”

Regarding the statement “More nuanced approaches are clearly needed”, please provide more specific directions for future research based on your main findings.

Thank you for a well performed study and well written manuscript. I would be happy to review the revised manuscript.

Sincerely,

J

**********

what does this mean?). If published, this will include your full peer review and any attached files.

If you choose “no”, your identity will remain anonymous but your review may still be made public.

**Do you want your identity to be public for this peer review?**  For information about this choice, including consent withdrawal, please see our Privacy Policy

Reviewer #1: No

Reviewer #2: No

**********

[NOTE: If reviewer comments were submitted as an attachment file, they will be attached to this email and accessible via the submission site. Please log into your account, locate the manuscript record, and check for the action link "View Attachments". If this link does not appear, there are no attachment files.]

To ensure your figures meet our technical requirements, please review our figure guidelines: https://journals.plos.org/plosone/s/figures

You may also use PLOS’s free figure tool, NAAS, to help you prepare publication quality figures: https://journals.plos.org/plosone/s/figures#loc-tools-for-figure-preparation.

NAAS will assess whether your figures meet our technical requirements by comparing each figure against our figure specifications.

# Author comment: Author response to Decision Letter 1

**DOI:** 10.1371/journal.pone.0358453.r002

9 Aug 2026

See attached file "Response to Reviewers." All responses to reviewer comments are in red text.

Attachment Submitted filename: Response to Reviewers.docx

# Peer review: Decision Letter 1

**DOI:** 10.1371/journal.pone.0358453.r003

**Authors:** Stanley Chinedu Eneh

19 Aug 2026

PONE-D-26-04064R1

Who is disrupting whom? Examining the nighttime activity patterns of humans and their dogs

PLOS One?

Dear Dr. Li,

Thank you for submitting your manuscript to PLOS One. After careful consideration, we feel that it has merit but does not fully meet PLOS One’s publication criteria as it currently stands. Therefore, we invite you to submit a revised version of the manuscript that addresses the points raised during the review process.

Please submit your revised manuscript by Oct 03 2026 11:59PM. If you will need more time than this to complete your revisions, please reply to this message or contact the journal office at plosone@plos.org. When you're ready to submit your revision, log on to https://www.editorialmanager.com/pone/ and select the 'Submissions Needing Revision' folder to locate your manuscript file.

Please include the following items when submitting your revised manuscript:

- A letter that responds to each point raised by the academic editor and reviewer(s). You should upload this letter as a separate file labeled 'Response to Reviewers'.
- A marked-up copy of your manuscript that highlights changes made to the original version. You should upload this as a separate file labeled 'Revised Manuscript with Track Changes'.
- An unmarked version of your revised paper without tracked changes. You should upload this as a separate file labeled 'Manuscript'.

If you would like to make changes to your financial disclosure, please include your updated statement in your cover letter. Guidelines for resubmitting your figure files are available below the reviewer comments at the end of this letter.

If applicable, we recommend that you deposit your laboratory protocols in protocols.io to enhance the reproducibility of your results. Protocols.io assigns your protocol its own identifier (DOI) so that it can be cited independently in the future. For instructions see: https://journals.plos.org/plosone/s/submission-guidelines#loc-laboratory-protocols. Additionally, PLOS One offers an option for publishing peer-reviewed Lab Protocol articles, which describe protocols hosted on protocols.io. Read more information on sharing protocols at https://plos.org/protocols?utm_medium=editorial-email&utm_source=authorletters&utm_campaign=protocols.

As the corresponding author, your ORCID iD is verified in the submission system and will appear in the published article. PLOS supports the use of ORCID, and we encourage all coauthors to register for an ORCID iD and use it as well. Please encourage your coauthors to verify their ORCID iD within the submission system before final acceptance, as unverified ORCID iDs will not appear in the published article. *Only*  the individual author can complete the verification step; PLOS staff *cannot*  verify ORCID iDs on behalf of authors.

We look forward to receiving your revised manuscript.

Kind regards,

Stanley Chinedu Eneh

Academic Editor

PLOS One

Journal Requirements:

1. If the reviewer comments include a recommendation to cite specific previously published works, please review and evaluate these publications to determine whether they are relevant and should be cited. There is no requirement to cite these works unless the editor has indicated otherwise.

2. Please review your reference list to ensure that it is complete and correct. If you have cited papers that have been retracted, please include the rationale for doing so in the manuscript text, or remove these references and replace them with relevant current references. Any changes to the reference list should be mentioned in the rebuttal letter that accompanies your revised manuscript. If you need to cite a retracted article, indicate the article’s retracted status in the References list and also include a citation and full reference for the retraction notice.

Additional Editor Comments:

Many thanks for your detailed response to our comments. The manuscript is much clearer as a result. From me, two very minor comments remain:

Lines 242-274: might benefit from some interpretation of what the lag times mean, as I think many people will be unfamiliar with this analysis. I appreciate this is included in the methods, but a little reminder here might do no harm!

Limitations: Given that the single survey was completed at the end of a four week period, this is likely to have introduced some recall bias which should be mentioned as a limitation - perhaps where you talk about use of a diary as an alternative?

[Note: HTML markup is below. Please do not edit.]

Reviewer's Responses to Questions

**Comments to the Author**

Reviewer #1: (No Response)

Reviewer #2: All comments have been addressed

**********

2. Is the manuscript technically sound, and do the data support the conclusions?

Reviewer #1: Yes

Reviewer #2: Yes

**********

3. Has the statistical analysis been performed appropriately and rigorously?

Reviewer #1: Yes

Reviewer #2: Yes

**********

4. Have the authors made all data underlying the findings in their manuscript fully available?

The PLOS Data policy

Reviewer #1: Yes

Reviewer #2: Yes

**********

5. Is the manuscript presented in an intelligible fashion and written in standard English?

Reviewer #1: Yes

Reviewer #2: Yes

**********

Reviewer #1: Many thanks for your detailed response to our comments. The manuscript is much clearer as a result. From me, two very minor comments remain:

Lines 242-274: might benefit from some interpretation of what the lag times mean, as I think many people will be unfamiliar with this analysis. I appreciate this is included in the methods, but a little reminder here might do no harm!

Limitations: Given that the single survey was completed at the end of a four week period, this is likely to have introduced some recall bias which should be mentioned as a limitation - perhaps where you talk about use of a diary as an alternative?

Reviewer #2: Dear authors,

Thank you for your thorough review of your manuscript! All my comments have been met.

Sincerely, J

**********

what does this mean?). If published, this will include your full peer review and any attached files.

If you choose “no”, your identity will remain anonymous but your review may still be made public.

**Do you want your identity to be public for this peer review?**  For information about this choice, including consent withdrawal, please see our Privacy Policy

Reviewer #1: No

Reviewer #2: No

**********

[NOTE: If reviewer comments were submitted as an attachment file, they will be attached to this email and accessible via the submission site. Please log into your account, locate the manuscript record, and check for the action link "View Attachments". If this link does not appear, there are no attachment files.]

To ensure your figures meet our technical requirements, please review our figure guidelines: https://journals.plos.org/plosone/s/figures

You may also use PLOS’s free figure tool, NAAS, to help you prepare publication quality figures: https://journals.plos.org/plosone/s/figures#loc-tools-for-figure-preparation.

NAAS will assess whether your figures meet our technical requirements by comparing each figure against our figure specifications.

# Author comment: Author response to Decision Letter 2

**DOI:** 10.1371/journal.pone.0358453.r004

28 Aug 2026

Reviewer #1: Many thanks for your detailed response to our comments. The manuscript is much clearer as a result. From me, two very minor comments remain:

Lines 242-274: might benefit from some interpretation of what the lag times mean, as I think many people will be unfamiliar with this analysis. I appreciate this is included in the methods, but a little reminder here might do no harm!

Thanks for this suggestion. We have added more explanation to make this section clearer for readers (lines 238-243).

Limitations: Given that the single survey was completed at the end of a four week period, this is likely to have introduced some recall bias which should be mentioned as a limitation - perhaps where you talk about use of a diary as an alternative?

We have added this to the limitations (lines 405-407).

Attachment Submitted filename: Response_to_Reviewers_auresp_2.docx

# Peer review: Decision Letter 2

**DOI:** 10.1371/journal.pone.0358453.r005

**Authors:** Stanley Chinedu Eneh

2 Sep 2026

Who is disrupting whom? Examining the nighttime activity patterns of humans and their dogs

PONE-D-26-04064R2

Dear Dr. Li,

We’re pleased to inform you that your manuscript has been judged scientifically suitable for publication and will be formally accepted for publication once it meets all outstanding technical requirements.

Within one week, you’ll receive an e-mail detailing the required amendments. When these have been addressed, you’ll receive a formal acceptance letter and your manuscript will be scheduled for publication.

An invoice will be generated when your article is formally accepted. Please note, if your institution has a publishing partnership with PLOS and your article meets the relevant criteria, all or part of your publication costs will be covered. Please make sure your user information is up-to-date by logging into Editorial Manager at Editorial Manager® and clicking the ‘Update My Information' link at the top of the page. For questions related to billing, please contact billing support.

If your institution or institutions have a press office, please notify them about your upcoming paper to help maximize its impact. If they’ll be preparing press materials, please inform our press team as soon as possible -- no later than 48 hours after receiving the formal acceptance. Your manuscript will remain under strict press embargo until 2 pm Eastern Time on the date of publication. For more information, please contact onepress@plos.org.

Kind regards,

Stanley Chinedu Eneh

Academic Editor

PLOS One

Additional Editor Comments (optional):

Reviewers' comments:

Reviewer's Responses to Questions

**Comments to the Author**

Reviewer #1: All comments have been addressed

**********

2. Is the manuscript technically sound, and do the data support the conclusions?

Reviewer #1: Yes

**********

3. Has the statistical analysis been performed appropriately and rigorously?

Reviewer #1: Yes

**********

4. Have the authors made all data underlying the findings in their manuscript fully available?

The PLOS Data policy

Reviewer #1: Yes

**********

5. Is the manuscript presented in an intelligible fashion and written in standard English?

Reviewer #1: Yes

**********

Reviewer #1: Many thanks, I have no further comments and I am happy to recommend this article for acceptance.

**********

what does this mean?). If published, this will include your full peer review and any attached files.

If you choose “no”, your identity will remain anonymous but your review may still be made public.

**Do you want your identity to be public for this peer review?**  For information about this choice, including consent withdrawal, please see our Privacy Policy

Reviewer #1: No

**********

# Editor report: Acceptance letter

**DOI:** 10.1371/journal.pone.0358453.r006

**Authors:** Stanley Chinedu Eneh

PONE-D-26-04064R2

PLOS One

Dear Dr. Li,

I'm pleased to inform you that your manuscript has been deemed suitable for publication in PLOS One. Congratulations! Your manuscript is now being handed over to our production team.

At this stage, our production department will prepare your paper for publication. This includes ensuring the following:

* All references, tables, and figures are properly cited

* All relevant supporting information is included in the manuscript submission,

* There are no issues that prevent the paper from being properly typeset

You will receive further instructions from the production team, including instructions on how to review your proof when it is ready. Please keep in mind that we are working through a large volume of accepted articles, so please give us a few days to review your paper and let you know the next and final steps.

Lastly, if your institution or institutions have a press office, please let them know about your upcoming paper now to help maximize its impact. If they'll be preparing press materials, please inform our press team within the next 48 hours. Your manuscript will remain under strict press embargo until 2 pm Eastern Time on the date of publication. For more information, please contact onepress@plos.org.

You will receive an invoice from PLOS for your publication fee after your manuscript has reached the completed accept phase. If you receive an email requesting payment before acceptance or for any other service, this may be a phishing scheme. Learn how to identify phishing emails and protect your accounts at https://explore.plos.org/phishing.

If we can help with anything else, please email us at customercare@plos.org.

Thank you for submitting your work to PLOS One and supporting open access.

Kind regards,

PLOS One Editorial Office Staff

on behalf of

Dr. Stanley Chinedu Eneh

Academic Editor

PLOS One
